# Combination S100A1 and ARC gene therapy as a treatment for Duchenne muscular dystrophy cardiomyopathy

**DOI:** 10.1172/jci.insight.204852

**Published:** 2026-06-09

**Authors:** David W. Hammers, Cora C. Hart, Eli Zerpa, Karen I. Laurent, Young il Lee, Meg M. Sleeper, H. Lee Sweeney

**Affiliations:** 1 Department of Pharmacology & Therapeutics and; 2 Myology Institute, University of Florida College of Medicine, Gainesville, Florida, USA.; 3 Small Animal Clinical Sciences, University of Florida College of Veterinary Medicine, Gainesville, Florida, USA.

**Keywords:** Cardiology, Muscle biology, Cardiovascular disease, Gene therapy, Neuromuscular disease

## Abstract

Duchenne muscular dystrophy (DMD) is a lethal pediatric striated muscle disease caused by loss of dystrophin for which there is no cure. Cardiomyopathy is the leading cause of death among individuals with DMD, and effective therapeutics to treat DMD cardiomyopathy are a major unmet clinical need. This work investigated adeno-associated viral (AAV) gene therapy approaches to treat DMD cardiomyopathy by overexpression of the calcium binding proteins S100A1 and apoptosis repressor with caspase recruitment domain (ARC). Using the severe D2*.mdx* mouse model of DMD, we identified that S100A1 gene therapy improves the diastolic dysfunction associated with DMD cardiomyopathy, whereas ARC gene therapy prolongs survival. The combination of S100A1 and ARC in a single bicistronic vector improves the long-term cardiac outcome and histopathology of D2.*mdx* mice and the development of heart failure caused by micro-dystrophin expression, and its safety was exhibited via intracoronary delivery in a canine model of DMD. In addition to robust cardiac benefits, S100A1-ARC gene therapy benefits D2.*mdx* skeletal muscle function and histopathology when driven by a striated muscle promoter. Together, these findings indicate that S100A1-ARC gene therapy represents an effective treatment for DMD cardiomyopathy and may have therapeutic benefits in treating other forms of cardiomyopathy and muscle pathologies.

## Abstract 2

<span>Cardiomyopathy is the leading cause of death in Duchenne muscular dystrophy. This work details a gene therapy that improves the outcomes for dystrophic hearts.</span>

## Graphical abstract

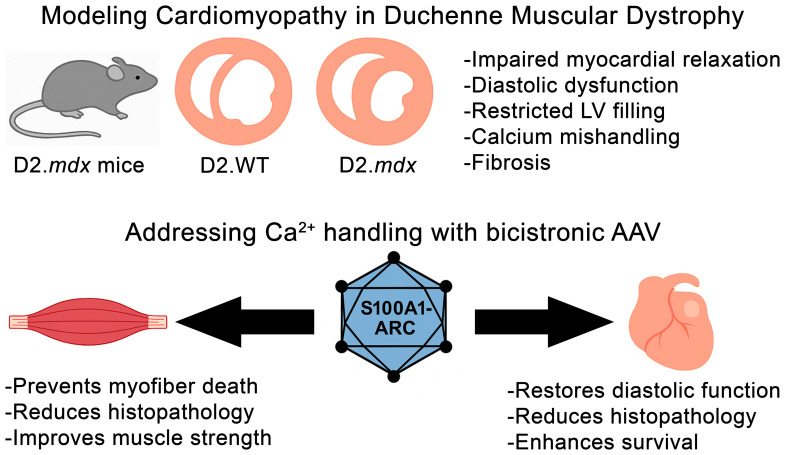

## Introduction

Duchenne muscular dystrophy (DMD) is a lethal X-linked pediatric muscle disease caused by *DMD* mutations that result in loss of dystrophin, a protein required for the stabilization of muscle during contractile activity (1, 2). Respiratory care advancements for DMD have increased the life expectancy for affected individuals (3, 4), and, as a consequence, cardiomyopathy has emerged as the leading cause of DMD mortality (5). Currently, effective therapeutics to combat dystrophic cardiomyopathy are a major unmet clinical need for individuals with DMD.

Prominent aspects of DMD cardiomyopathy include electrical conduction abnormalities and arrhythmic events that can be detected early in life (4, 6–8) and the gradual progression to dilated cardiomyopathy (DCM) in the second to third decade of life (9). A feature of DMD cardiomyopathy receiving increased recognition is a prolonged phase of left ventricular (LV) restriction and pronounced diastolic dysfunction that precedes overt decline of systolic function (10–13). Although stroke volume (SV) deficits and LV wall movement abnormalities have been reported in younger boys with DMD for decades (8), advanced echocardiography and cardiac magnetic resonance techniques yield data clearly depicting this restrictive phenotype in patients with DMD prior to ejection fraction (EF) decline (11, 13–15). Su et al. (11) brought specific attention to this LV restriction in DMD, coined as “tonic contraction,” and the recent work of Starnes et al. (12) demonstrates clear impairments of DMD LV filling using cardiac magnetic resonance. In agreement with these clinical findings, diastolic impairments are also evident in mouse and dog models of DMD (16–19).

Mechanisms contributing to the development of DMD cardiomyopathy largely converge on the central hypothesis that dystrophin loss causes impaired cardiomyocyte calcium handling (20–22). This model entails persistently high intracellular calcium concentrations caused by sarcolemma tears, dysfunctional cation channels, sarcoplasmic reticulum leakage, and/or insufficient removal of calcium from the cytosol. These features result in a myriad of detrimental consequences including, but not limited to, impaired relaxation, arrhythmogenic events, protease activation, mitochondrial dysfunction, and cell death (17, 22–25). To address this pathogenic calcium dysregulation in dystrophic cardiomyocytes, several strategies have been employed to restore calcium homeostasis, including increasing sarcoplasmic/ER calcium ATPase (SERCA) levels/activity (26–28), addressing sarcoplasmic reticulum calcium leak through ryanodine receptors (29, 30), blocking calcium-permeable cation channels (31, 32), and preventing sarcolemmal tears with membrane sealants (33, 34).

The purpose of the current study was to investigate the impact of S100A1, a calcium binding protein that exerts several benefits in the context of calcium dysregulation during heart failure (35), and apoptosis repressor with caspase recruitment domain (ARC), an anti-apoptotic protein that improves cardiomyocyte survival during myocardial infarction (36–38), as gene therapies for DMD cardiomyopathy. Herein, we provide the time course of D2.*mdx* cardiac progression to a restrictive phenotype with severe diastolic dysfunction. This phenotype was effectively prevented by adeno-associated virus–mediated (AAV-mediated) cardiac S100A1 overexpression. AAV-mediated cardiac ARC overexpression, on the other hand, did not largely affect LV restriction, but prolonged the survival of D2.*mdx* mice. Furthermore, a bicistronic AAV containing both S100A1 and ARC improved long-term cardiac outcomes of D2.*mdx* mice, prevented heart failure associated with micro-dystrophin (μDys), and exhibited safety and evidence of cardiac improvements in the golden retriever muscular dystrophy (GRMD) canine model of DMD. Together, these findings indicate that the combinational delivery of S100A1 and ARC utilizing a bicistronic AAV approach represents an effective gene therapy strategy for the treatment of DMD cardiomyopathy.

## Results

### Progressive diastolic dysfunction and LV restriction in D2.mdx mice.

The mild progressing course of disease in traditional *mdx* mouse models of DMD (on C57-based genetic backgrounds) requires advanced aging or use of stress-inducing stimuli to elicit functional deficits (39, 40), which has limited the potential for mechanistic insights and therapeutic development for DMD cardiomyopathy. The backcrossing of the dystrophin nonsense *mdx* mutation onto the DBA/2J genetic background (known as the D2.*mdx* mouse) results in more severe muscle pathology than that exhibited by C57-based *mdx* mouse lines (41, 42). We have previously described and utilized this emerging D2.*mdx* model to evaluate translational therapeutics for the severe pathological features of DMD skeletal muscle (16, 43–45). The increased disease severity on the DBA/2J background is attributed to a deletion in the latent TGF-β binding protein 4 (LTBP4) polyproline-rich hinge (46, 47), which makes the DBA/2J variant of the LTBP4 hinge closer in size to that of other mammalian species, including rats, pigs, dogs, and humans (Supplemental Figure 1; supplemental material available online with this article; https://doi.org/10.1172/jci.insight.204852DS1), and more susceptible and more susceptible to activating TGF-β (47). To gain a better insight into this murine model’s utility for understanding and testing therapeutic interventions for DMD cardiomyopathy, we performed a comprehensive evaluation of cardiomyopathy development in D2.*mdx* mice using ECG, echocardiography, and histology.

Electrical abnormalities detected using ECG are typically the first clinical symptoms of DMD cardiomyopathy (6–8); therefore, we analyzed ECGs from DBA/2J WT (D2.WT) and D2.*mdx* mice from 2 to 12 months of age. Similar to observations in younger boys with DMD, ECG abnormalities are evident in D2.*mdx* hearts as early as 2 months of age (Supplemental Table 1). This includes significant genotype effects for prolonged QT, JT, and T_peak_ to T_end_ intervals, as well as increased T wave amplitude, which are indicative of impaired LV repolarization. These data confirm that, like boys with DMD, D2.*mdx* mice exhibit electrical abnormalities early in life.

**Figure 1:**
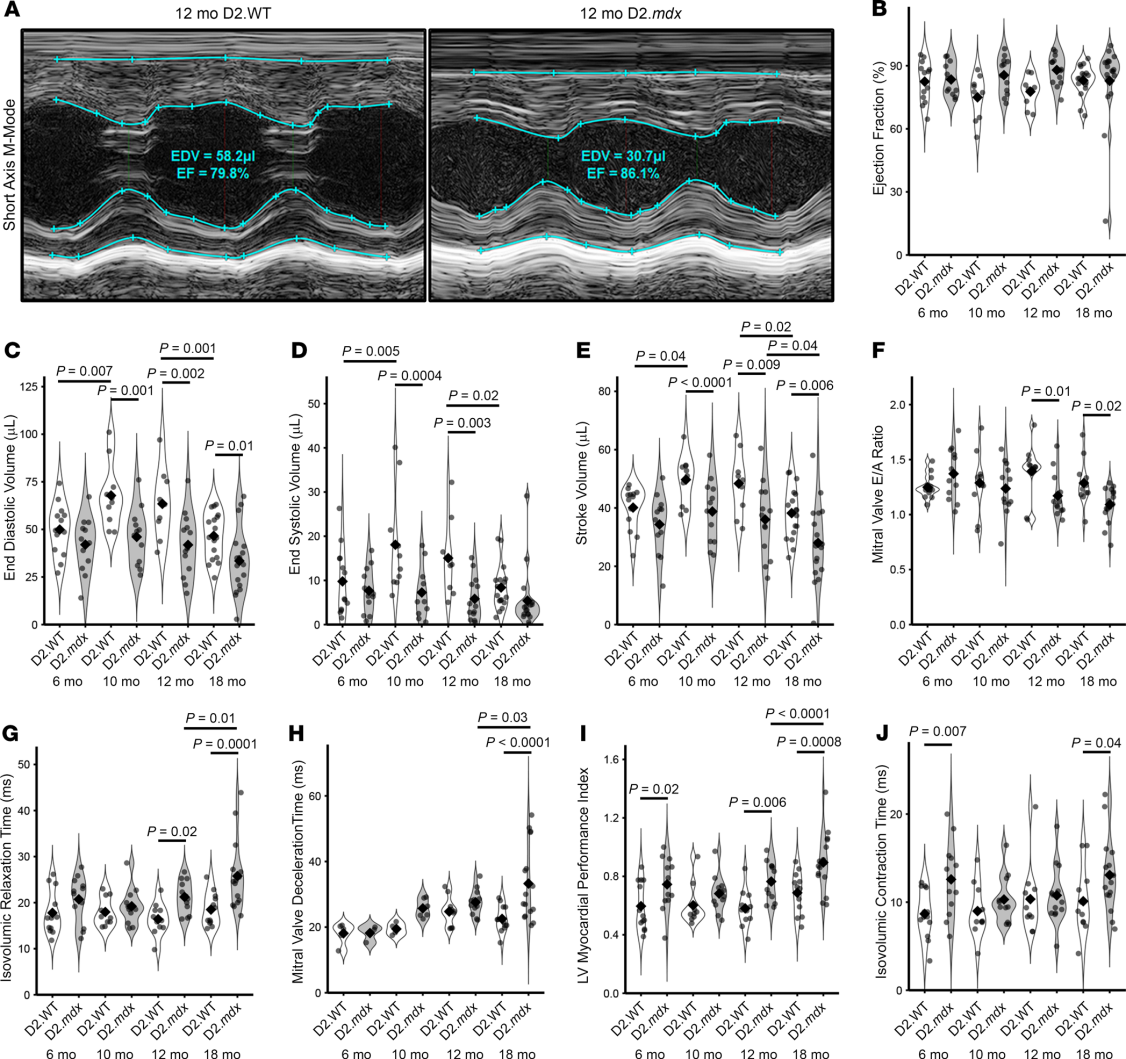
Progressive diastolic dysfunction and LV restriction in D2.*mdx* mice. Echocardiography was performed on D2.WT (WT; *n* = 9–17) and D2.*mdx* (*mdx*; *n* = 12–18) mice at the ages of 6, 10, 12, and 18 months. (**A**) Short-axis M-mode was used to measure the left ventricular (LV) parameters of (**B**) ejection fraction, (**C**) end diastolic volume, (**D**) end systolic volume, and (**E**) stroke volume. Pulsed wave Doppler was used to determine (**F**) mitral valve E/A ratios, (**G**) LV isovolumic relaxation time, (**H**) mitral valve deceleration time, (**I**) LV myocardial performance index, and (**J**) LV isovolumic contraction time. Data are displayed as violin plots (individual values indicated by circles; means indicated by black diamonds) and were analyzed using 2-factor ANOVA (age and genotype effects) followed by multiple *t* tests with Bonferroni’s correction (α = 0.05; significant *P* values are indicated).

Diastolic dysfunction and restrictive LV filling are becoming more frequently reported aspects of DMD cardiomyopathy prior to the onset of systolic deficits and DCM development (10–13, 15). To analyze the emergence of these features in D2.*mdx* mice, we performed echocardiography on D2.WT and D2.*mdx* mice at 6, 10, 12, and 18 months. M-mode short axis (Figure 1A) analyses revealed no age- or genotype-associated differences in EF (Figure 1B); however, D2.*mdx* mice exhibited progressive declines in end diastolic volume (EDV; Figure 1C), end systolic volume (ESV; Figure 1D), and SV (Figure 1E) beginning at 10 months, with significant age and genotype effects. Four-chamber pulsed-wave Doppler measurements revealed significant D2.*mdx-*associated reductions in mitral valve (MV) E/A ratio (Figure 1F), a key indicator of diastolic dysfunction. Significant age- and genotype-associated increases in other measures of impaired LV relaxation and diastolic filling, including LV isovolumic relaxation time (Figure 1G), MV deceleration time (Figure 1H), and LV myocardial performance index (Figure 1I), were also found. Additionally, LV isovolumic contraction time was significantly increased in D2.*mdx* mice (Figure 1J), suggesting that defects in LV contractility, pressure development, and/or aortic valve opening may be aspects of cardiomyopathy in the D2.*mdx* mouse. These measures of impaired D2.*mdx* diastolic function were not due to faster heart rates than WT counterparts (Supplemental Figure 2A). Although evidence of LV wall thinning at diastole were not observed to confirm progression to DCM in those mice (Supplemental Figure 2, B and C), individual instances of markedly reduced EF may indicate that systolic failure begins around 18 months in this model. Although some features of LV diastolic dysfunction and restriction may be interpreted as development of cardiac hypertrophy, we identified no evidence of D2.*mdx-*associated cardiac enlargement at the organ or cardiomyocyte level in 12-month-old mice (Supplemental Table 2). Furthermore, we identified many of these LV restriction phenotypes in historical data from GRMD dogs, an established large animal model to study DMD cardiomyopathy (17, 48, 49). This analysis revealed affected GRMD dogs exhibit reduced LV end diastolic parameters, SVs, and MV E/A ratios compared with normal and carrier canines (Supplemental Table 3). These findings indicate that diastolic dysfunction with LV restriction is a translational feature of DMD cardiomyopathy that is prominently exhibited in the D2.*mdx* mouse model of DMD.

**Figure 2:**
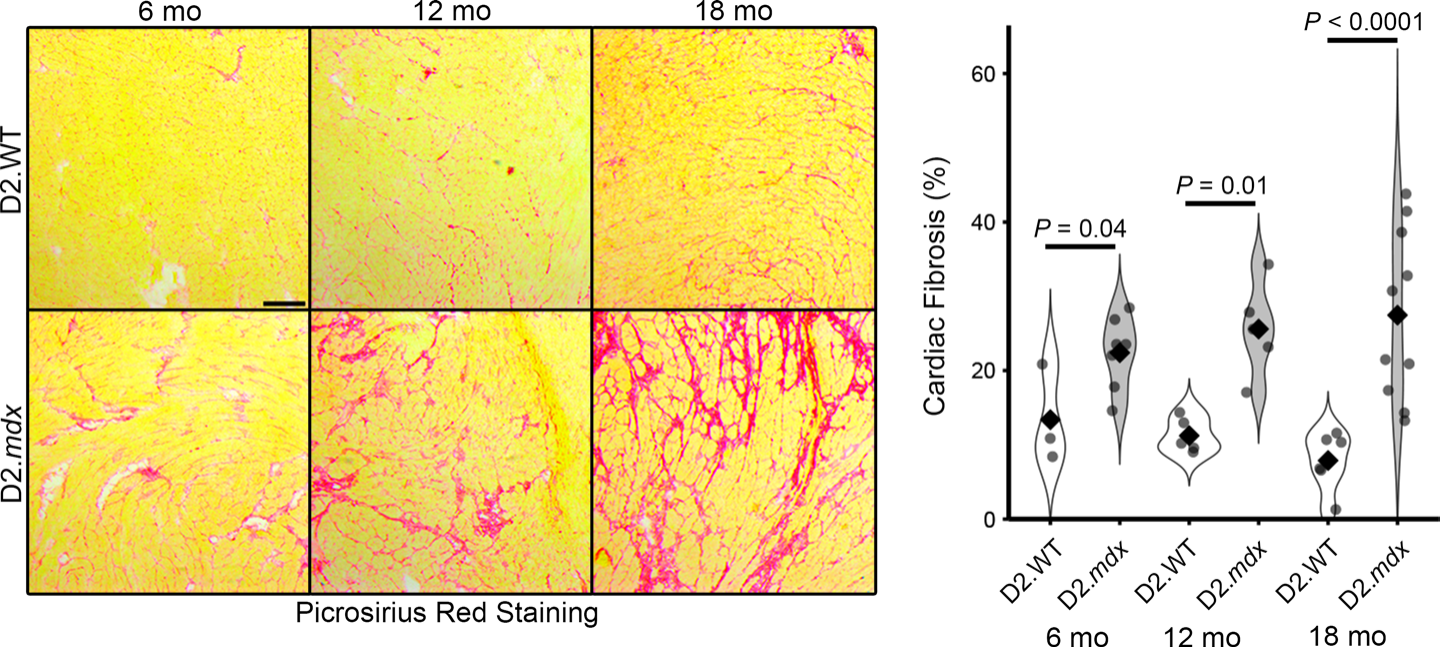
Progressive cardiac fibrosis in D2.*mdx* mice. Cardiac fibrosis was visualized and quantified using Picrosirius red staining of heart sections from 6-, 12-, and 18-month-old mice of each genotype (scale bar: 100 μm). Data are displayed as violin plots (individual values indicated by circles; means indicated by black diamonds) and were analyzed using 2-factor ANOVA (age and genotype effects) followed by multiple *t* tests with Bonferroni’s correction (α = 0.05; significant *P* values are indicated).

Similar to the severe histopathology exhibited by D2.*mdx* skeletal muscle (44), the hearts of these mice also showed increased fibrosis and tissue pathology, as demonstrated by Picrosirius red staining (Figure 2). This is consistent with features found in boys with DMD and most preclinical models of DMD (17, 50–53). Because cardiac fibrosis is a potential cause of LV restriction via impairment of proper cardiomyocyte elongation during diastole, we employed the cardiac myosin inhibitor mavacamten (54), also known as MYK-461, to answer whether the reduced diastolic function and LV filling of D2.*mdx* mice is due to cardiomyocyte-intrinsic (i.e., impaired relaxation) or cardiomyocyte-extrinsic (i.e., fibrosis) factors. First, we confirmed that D2.WT mice were responsive to oral dosing of mavacamten (2.5 mg/kg), as evidenced by a significant decline in fractional shortening 3 hours after administration (Supplemental Figure 3A). This treatment resulted in a modest but nonsignificant increase in D2.WT EDV (Supplemental Figure 3B). We next repeated this experiment in 10-month-old D2.*mdx* mice, with the addition of a 3-day posttreatment washout period (diagramed in Supplemental Figure 3C). Similar to the effect seen in D2.WT mice, D2.*mdx* hearts exhibited a significant reduction in fractional shortening 3 hours after mavacamten dosing, which was completely reversed after washout (Supplemental Figure 3D). In contrast to D2.WT hearts, however, mavacamten caused D2.*mdx* hearts to show a significant approximately 68% increase in EDV, which was also reversed by washout (Supplemental Figure 3E). These findings support the hypothesis that the restricted LV filling of D2.*mdx* cardiomyopathy involves cardiomyocyte-intrinsic intracellular mechanisms, such as elevated resting calcium levels (16, 55), which potentially represent a targetable feature to treat DMD cardiomyopathy.

### S100A1 treatment alleviates LV restriction and ARC prolongs lifespan in D2.mdx mice.

Multiple gene therapies have been designed to address calcium handling issues in DMD cardiomyopathy by increasing SERCA activity (26–28). Although effective in mitigating elevated cytosolic calcium concentrations, these strategies also increase ATP demands in already energetically challenged dystrophic cardiomyocytes (20). As an alternative approach to address this calcium dysregulation with gene therapy, we sought to individually test the efficacy of overexpressing either S100A1, a pleiotropic calcium-binding protein that exerts several benefits in failing cardiomyocytes (35), or ARC, a protein that represses calcium dysregulation’s ultimate downstream effect of cell death (36, 37, 56).

**Figure 3:**
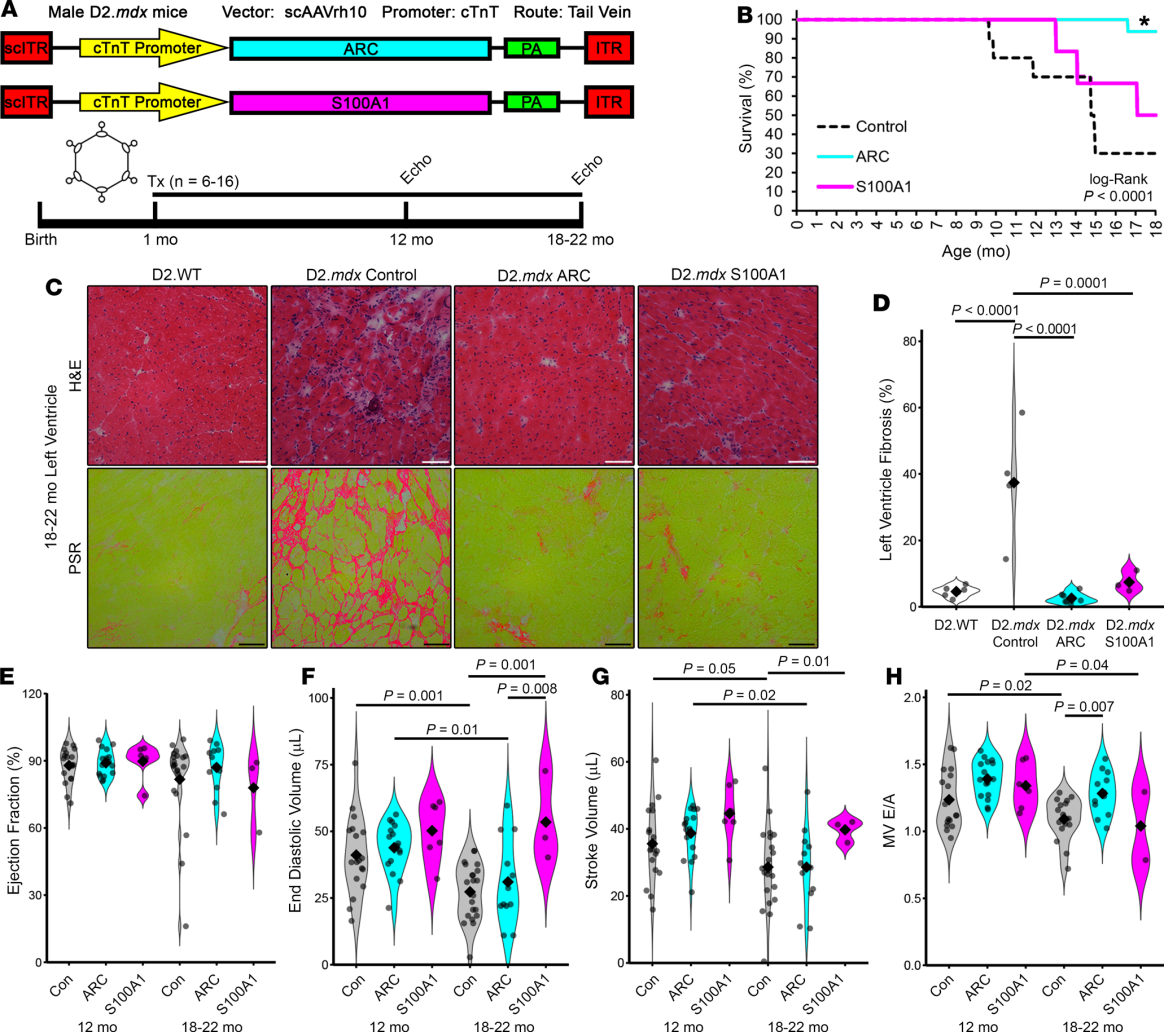
Therapeutic effect of S100A1 and ARC in D2.*mdx* mouse hearts. (**A**) Male D2.*mdx* received systemic treatment with 6.33 × 10^13^ gc/kg scAAVrh10.cTnT vector containing the coding sequence for either S100A1 (*n* = 6) or ARC (*n* = 16) at 1 month of age and underwent echocardiography at the 12-month and 18–22-month time points. Control D2.*mdx* mice (Con; *n* = 10) were included in this cohort for survival analysis, whereas additional control values are included in echocardiography measurements. (**B**) Mouse survival is depicted in a Kaplan-Meier plot. (**C**) Representative images of LV histopathology as visualized by H&E and Picrosirius red (PSR) staining (scale bar: 100 μm) and (**D**) fibrosis quantification. Echocardiography determined values for (**E**) ejection fraction, (**F**) end diastolic volume, (**G**) stroke volume, and (**H**) mitral valve (MV) E/A ratio for 12-month and 18–22-month time points. Data are displayed as (**B**) survival curve and (**D**–**H**) violin plots (individual values indicated by circles; means indicated by black diamonds). Data were analyzed using (**A**) Kaplan-Meier estimate followed by Wilcoxon’s nonparametric comparison test (α = 0.05; *P < 0.0001 vs. Con survival) and (**D**–**H**) 1-factor ANOVA (age and treatment effects) followed by multiple *t* tests with Bonferroni’s correction (α = 0.05; *P < 0.05 vs. age-matched Con values; significant *P* values are indicated).

To confirm that S100A1 and/or ARC are not naturally upregulated during dystrophic cardiomyopathy, immunoblotting for these proteins in 12-month-old D2.*mdx* hearts revealed trends of downregulation relative to age-matched WT levels (Supplemental Figure 4). To test the hypothesis that overexpression of each of these proteins would benefit dystrophic hearts, we developed individual self-complementary AAV vectors containing codon-optimized murine ARC and S100A1 driven by the cardiac troponin T (cTnT; *Tnnt2* gene) promoter (57) and packaged in AAVrh10 serotype vector (58, 59). These vectors were administered to 1-month-old male D2.*mdx* mice via tail vein injections using a dose of 6.33 × 10^13^ gc/kg (experiment depicted in Figure 3A), with echocardiography performed at 12 months and 18–22 months (endpoint determined when S100A1 treatment mice reached the point of mean survival). Both transgenes extended mean survival time beyond the course of control D2.*mdx* mice; however, this effect was significant with ARC treatment (Figure 3B). S100A1 and ARC also improved LV histopathology and reduced cardiac fibrosis compared with control hearts (Figure 3, C and D), in agreement with the observed survival benefits. Although echocardiography revealed no changes to EF across ages or treatments (Figure 3E), significant effects were observed in the key diastolic function parameters of EDV, SV, and MV E/A ratio (Figure 3, F, G, and H) at the older time point. Notably, S100A1 treatment prevents the decline of EDV and SV associated with D2.*mdx* cardiomyopathy, whereas ARC treatment preserves MV E/A ratio. These data indicate that cardiac expression of both ARC and S100A1 are beneficial for the treatment of D2.*mdx* cardiomyopathy with therapeutically distinct outcomes.

### Bicistronic S100A1-ARC vector improves D2.mdx cardiac phenotype.

**Figure 4:**
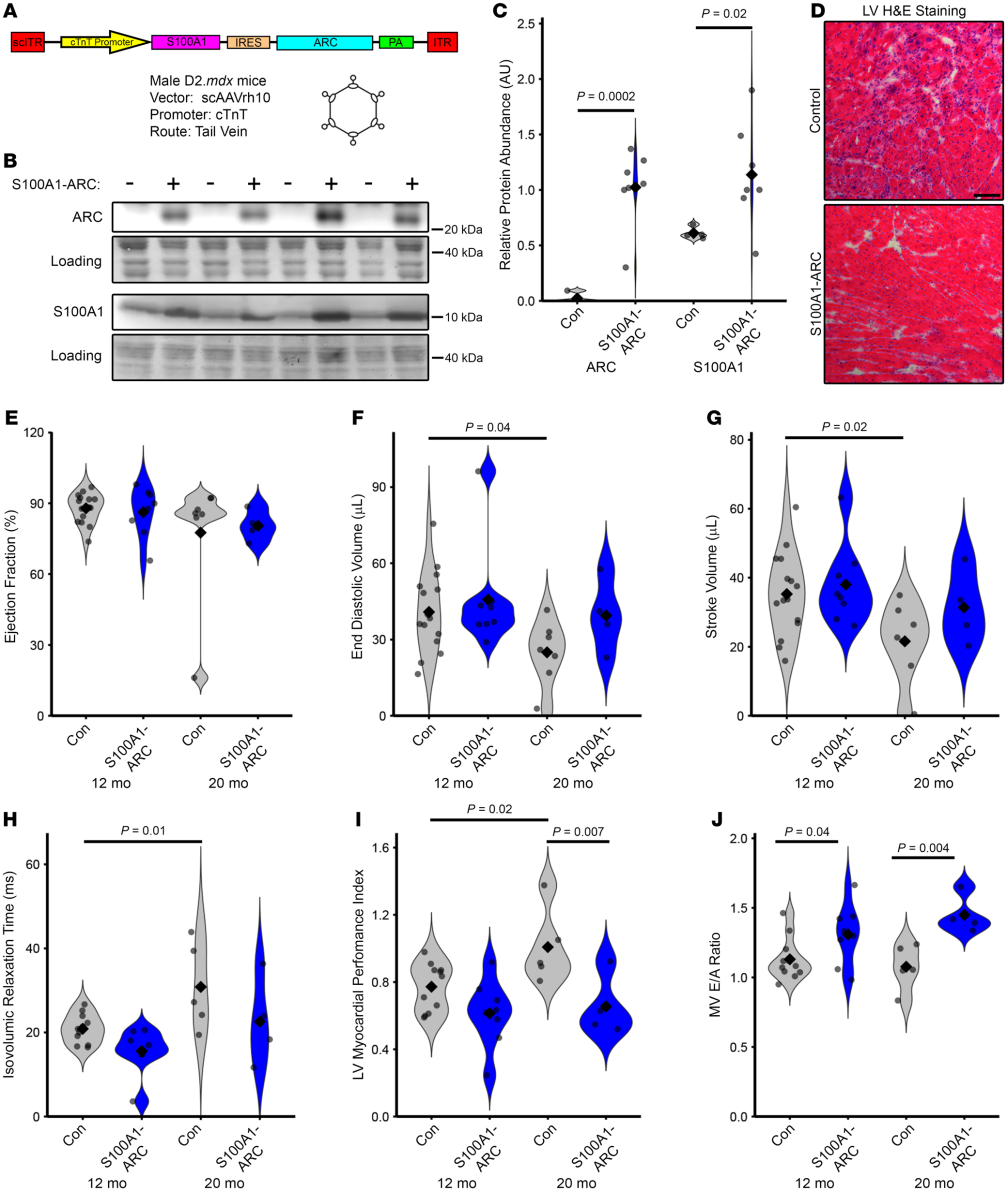
Bicistronic S100A1-ARC provides long-term benefits to D2.*mdx* cardiac function. (**A**) Male D2.*mdx* mice received control (Con; *n* = 12) or scAAVrh10.cTnT.S100A1-ARC (S100A1-ARC; *n* = 8) treatments at 1 month of age and underwent echocardiography at 12 and 20 months. (**B** and **C**) Cardiac ARC and S100A1 protein levels were assessed at study endpoint. (**D**) Representative images of left ventricle (LV) histopathology are provided, as analyzed by H&E staining (scale bar: 100 μm). Echocardiography measures of (**E**) ejection fraction, (**F**) end diastolic volume, (**G**) stroke volume, (**H**) isovolumic relaxation time, (**I**) LV myocardial performance index, and (**J**) mitral valve (MV) E/A ratios are shown for both 12-month and 20-month time points. Data are displayed as violin plots (individual values indicated by circles; means indicated by black diamonds) and were analyzed using (**C**) 2-tailed Welch’s *t* test (α = 0.05; *P < 0.05 vs. Con values) and (**E**–**J**) 2-factor ANOVA (age and treatment effects) followed by multiple *t* tests with Bonferroni’s correction (α = 0.05; *P < 0.05 vs. age-matched Con values; significant *P* values are indicated).

Given the improvements in diastolic function incurred by S100A1 overexpression and the survival benefit of ARC overexpression, we sought to combine these therapeutic transgenes into a single bicistronic vector utilizing an internal ribosomal entry site (60). The canine versions of these transgenes were used in this self-complementary therapeutic construct, which was also driven by the cTnT promoter and packaged in AAVrh10 vector (Figure 4A), to support further translational studies in larger mammals. Systemic treatment of this vector at a dose of 2 × 10^14^ gc/kg (16, 61) achieved approximately 10-fold elevation of ARC levels and approximately 2-fold elevation of S100A1 levels in D2.*mdx* mice that were injected at 1 month of age (Figure 4, B and C). Expression of canine S100A1 and ARC in this manner did not result in anti-transgene immune responses, as predicted by the high homology between murine and canine S100A1 (94%) and ARC (78%; primary differences in repetitive proline-glutamic acid–rich C-terminal tail). At the study’s endpoint (20 months), S100A1-ARC–treated hearts exhibited fewer signs of histopathology, including better cardiomyocyte organization and inflammation/infiltration (Figure 4D) and significantly reduced fibrosis (Supplemental Figure 5) than control D2.*mdx* hearts. Consistent with these findings and in agreement with the individual transgene treatments, this bicistronic vector had no effect on EF (Figure 4E) but did significantly reduce the progressive EDV and SV decline (Figure 4, F and G), LV isovolumic relaxation time elongation (Figure 4H), and worsening of LV myocardial performance index (Figure 4I). Additionally, the MV E/A ratio was significantly higher at both time points in S100A1-ARC–treated hearts (Figure 4J). It is important to note that although the S100A1-ARC treatment group underwent longitudinal measures in this study, no mice in the 12-month control group survived to the 20-month time point. We therefore utilized D2.*mdx* mice that survived to this time point for age-matched control data. These findings indicate that a bicistronic vector consisting of S100A1 and ARC provides substantial benefits to the dystrophic myocardium, particularly for the improvement of diastolic function.

### S100A1-ARC treatment prevents μDys-associated heart failure.

**Figure 5:**
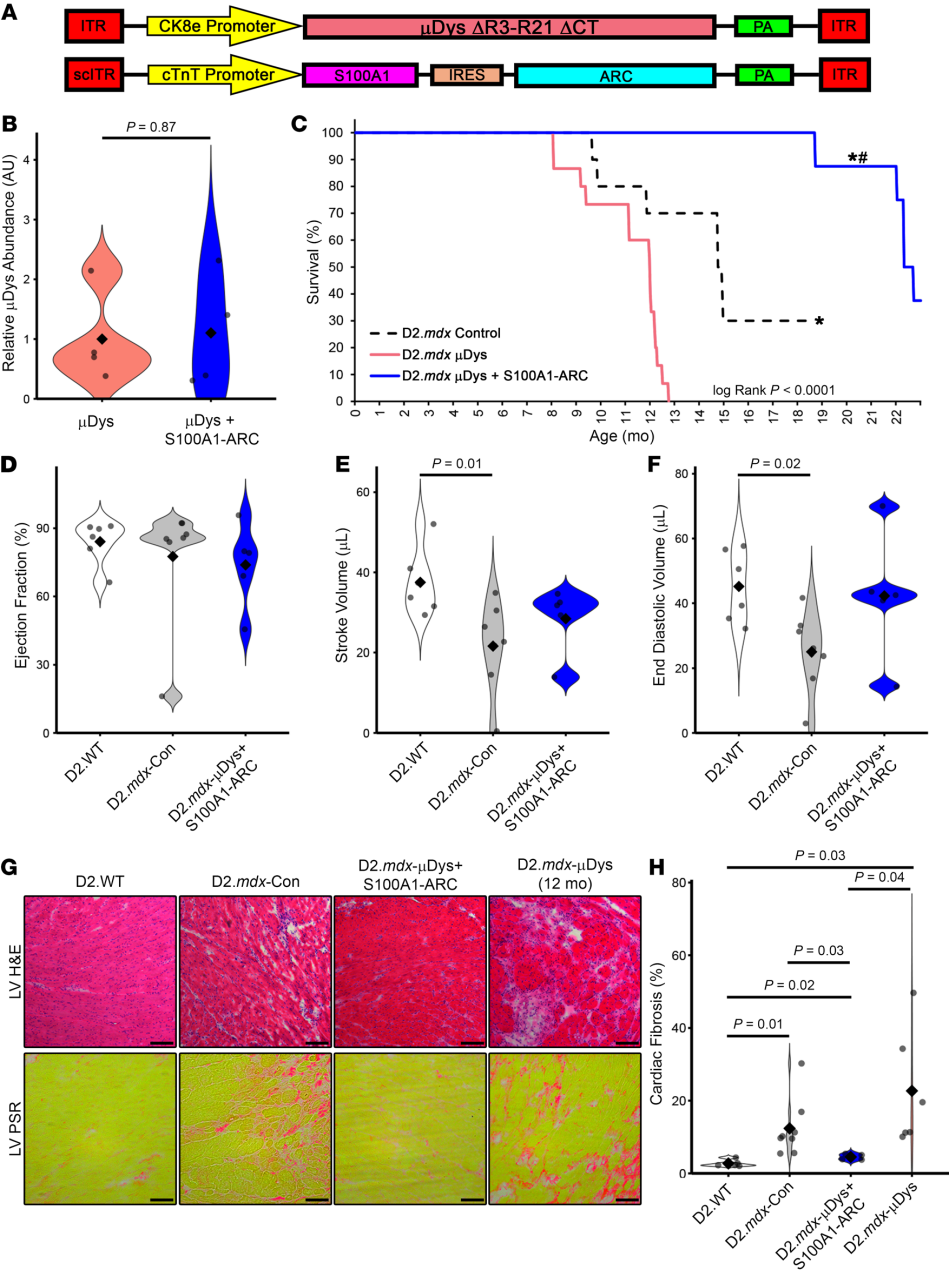
S100A1-ARC gene therapy prevents micro-dystrophin cardiotoxicity. (**A**) Male D2.*mdx* mice were treated with the ΔR3-R21ΔCT micro-dystrophin (μDys) gene therapy with (*n* = 8) and without (*n* = 15) cotreatment with the bicistronic cardiac S100A1-ARC vector at 1 month of age and were evaluated for survival. (**B**) Cardiac expression of μDys was measured by immunoblotting at respective humane endpoints of the study. (**C**) Survival analysis reveals significant life-extension by cotreatment with S100A1-ARC. Echocardiographic measures of (**D**) ejection fraction, (**E**) stroke volume, and (**F**) end diastolic volume are shown for 20–22-month-old D2.WT (*n* = 6) mice, 20-month-old control D2.*mdx* (*mdx-*Con; *n* = 7) mice, and 22-month-old D2.*mdx* mice that received both micro-dystrophin and S100A1-ARC treatments at 1 month (*mdx*-μDys+S100A1-ARC; *n* = 5). (**G**) Representative H&E and Picrosirius red (PSR) stained images of left ventricles (LV) from D2.WT, D2.*mdx*-Con, D2.*mdx*- μDys+S100A1-ARC, and 12-month-old D2.*mdx*-μDys mice only (reached humane endpoint; scale bar: 100 μm) demonstrate differential histopathology across groups. (**H**) Cardiac fibrosis was quantified from PSR-stained samples. Data are displayed as (**B** and **D**–**F**) violin plots with individual values indicated by circles and (**C**) a survival curve. Data were analyzed using (**B**) a 2-tailed Welch’s *t* test (α = 0.05; *P* value indicated), (**C**) a Kaplan-Meier estimator analysis (α = 0.05), and (**D**–**F**) 1-factor ANOVA followed by multiple *t* tests with Bonferroni’s correction (α = 0.05; *P < 0.05 vs. WT values). Control D2.*mdx* survival data in **C** are also reported in Figure 3B.

Systemic μDys gene therapies are in the clinic to treat DMD-affected individuals with a miniaturized dystrophin transgene designed to be expressed in all striated muscles (62). In previous work, we identified that specific μDys designs have the ability to promote cardiac dysfunction and premature death in D2.*mdx* mice, whereas other versions of these human μDys transgenes were either beneficial or had no effect on long-term cardiac outcomes (16). In particular, the lethality caused by the ΔR3-R21 ΔCT μDys (16, 63) at approximately 11 months after treatment has been confirmed to be caused by heart failure, as determined by heart failure gene expression markers of increased *Nppb* (Supplemental Figure 6A), decreased *Myh6* (Supplemental Figure 6B), and increased *Myh7* and *Myh7/Myh6* ratios (Supplemental Figure 6, C and D). To evaluate whether S100A1-ARC treatment can rescue this phenotype, we treated 1-month-old D2.*mdx* mice with ΔR3-R21 ΔCT μDys alone or in combination with S100A1-ARC (Figure 5A). Although there was no effect of this combination on μDys content (Figure 5B and Supplemental Figure 6E), ARC and S100A1 transgene levels were robustly expressed in only the dual vector treatment group (Supplemental Figure 6E). Immunofluorescence confirmed both μDys and ARC are uniformly expressed across combination-treated cardiomyocytes (Supplemental Figure 6F). Importantly, S100A1-ARC treatment significantly increased mouse survival from a mean survival time of 336 days to 625 days (Figure 5C). Because age-matched μDys-only mice were not available for comparison of heart function, we compared echocardiography data from 22-month-old μDys+S100A1-ARC–treated mice to those from 20–22-month-old D2.WT and 20-month-old D2.*mdx* groups. This comparison revealed no significant differences in EF across the groups, whereas SV and EDV from the μDys+S100A1-ARC group more closely resembled D2.WT values than those of untreated D2.*mdx* mice (Figure 5, D–F). Cardiac histopathology was also markedly improved in the combination treatment group, as shown by both H&E and Picrosirius red staining (Figure 5, G and H). These findings demonstrate that the S100A1-ARC bicistronic vector prevents the heart failure associated with cardiotoxic μDys transgenes and may represent an adjunct therapeutic, if μDys treatment promotes similar forms of heart failure in the clinic.

### S100A1-ARC gene therapy safety in GRMD canine model of DMD.

**Figure 6:**
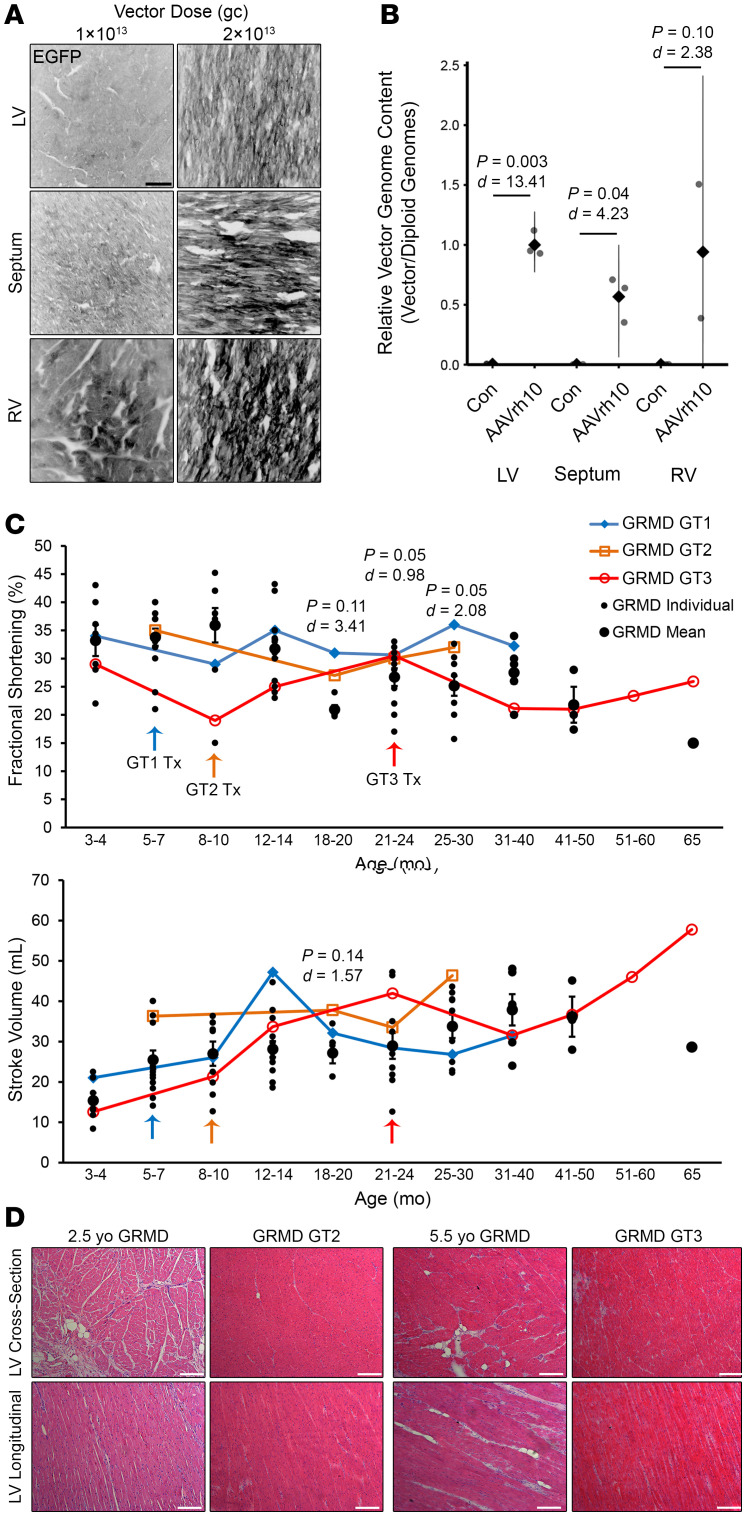
S100A1-ARC gene therapy in GRMD canines. (**A**) Representative epifluorescence images of EGFP expression in the left ventricle (LV), septum, and right ventricle (RV) of canine hearts 1 week after intracoronary delivery of 1 × 10^13^ or 2 × 10^13^ gc of AAVrh10.EGFP. (**B**) AAV vector genomes were quantified in the LV, septum, and RV of GRMD hearts after intracoronary treatment with 5 × 10^13^ gc of AAVrh10 vector. Data are displayed as violin plots (individual values indicated by circles; means indicated by black diamonds) and were analyzed using 2-tailed Welch’s *t* test (α = 0.05; *P* value and effect size indicated). (**C**) Fractional shortening and stroke volume measurements, as determined by echocardiography, from S100A1-ARC gene therapy–treated GRMD dogs (GT1 in blue, GT2 in orange, and GT3 in red; color-matched arrows indicate respective age of treatment). Historical GRMD values from the colony for each age range are shown in black (means as large circles with SEM shown; individual values as small circles). Age ranges having 2 or more GT values were analyzed using 2-tailed Welch’s *t* test (α = 0.05; *P* value and effect size indicated). (**D**) Representative H&E staining of LV cross-sections and longitudinal sections from S100A1-ARC–treated GRMD GT2 and GT3 with comparably age-matched control GRMD samples (scale bar: 100 μm).

We next sought to test the safety of myocardial S100A1-ARC expression in the GRMD canine model of DMD. Prior to S100A1-ARC development, we investigated AAV serotypes capable of efficient transduction of canine hearts via i.v. administration. Although AAV9 exhibited lower transduction than AAV8 in the initial testing (Supplemental Figure 7A), we identified AAVrh10 as a vector that efficiently transduces canine striated muscle at an i.v. delivered dose of 5 × 10^12^ gc/kg compared with AAV8 and AAVrh8 (Supplemental Figure 7, B and C). To further reduce amounts of vector required to specifically transduce canine hearts with therapeutic AAVs, we investigated the transduction efficiency of intracoronary AAV delivery, which revealed high transduction efficiency of the LV, septum, and right ventricle at doses of 2 × 10^13^ gc and 5 × 10^13^ gc of total vector delivery (Figure 6, A and B). Ensuing therapeutic treatments of scAAVrh10.cTnT.S100A1-ARC therefore utilized 5 × 10^13^ gc of vector delivered via intracoronary injections.

The bicistronic scAAVrh10.cTnT.S100A1-ARC vector was delivered to 3 GRMD dogs: GT1 at 5 months, GT2 at 10 months, and GT3 at 24 months. Because GRMD cardiomyopathy is progressive with age (17, 48), we chose this spectrum of treatment ages to verify the safety of vector delivery across ages representing different degrees of preexisting cardiac pathology. All treatments were well tolerated, and no overt signs of cardiomyopathy were exhibited during the course of this study, which was followed for years without signs of safety concern from the vector treatment. The longevity and eventual endpoints of these dogs are summarized in Supplemental Table 4.

The low sample size of this safety evaluation limited interpretation of efficacy from this treatment. However, echocardiography data from the treated canines of this group compared with historical age-matched data from the colony revealed large effect sizes for fractional shortening improvements at 18–20, 21–24, and 25–30 months and SV increases at 18–20 months for GT1 and GT2 (Figure 6C). In agreement with possible functional benefits associated with these GRMD treatments, tissue sections of GT2 and GT3 LV samples exhibited markedly reduced signs of histopathology compared with comparably age-matched untreated GRMD samples (Figure 6D). Immunofluorescence confirmed both the loss of dystrophin staining in GRMD LV sections and the persistence of ARC transgene in S100A1-ARC-treated LV (Supplemental Figure 8). Together, these data provide evidence of both safety and improved cardiac phenotype by intracoronary delivery of S100A1-ARC gene therapy in the GRMD heart.

### Skeletal muscle phenotypes improved by striated muscle expression of S100A1-ARC.

**Figure 7:**
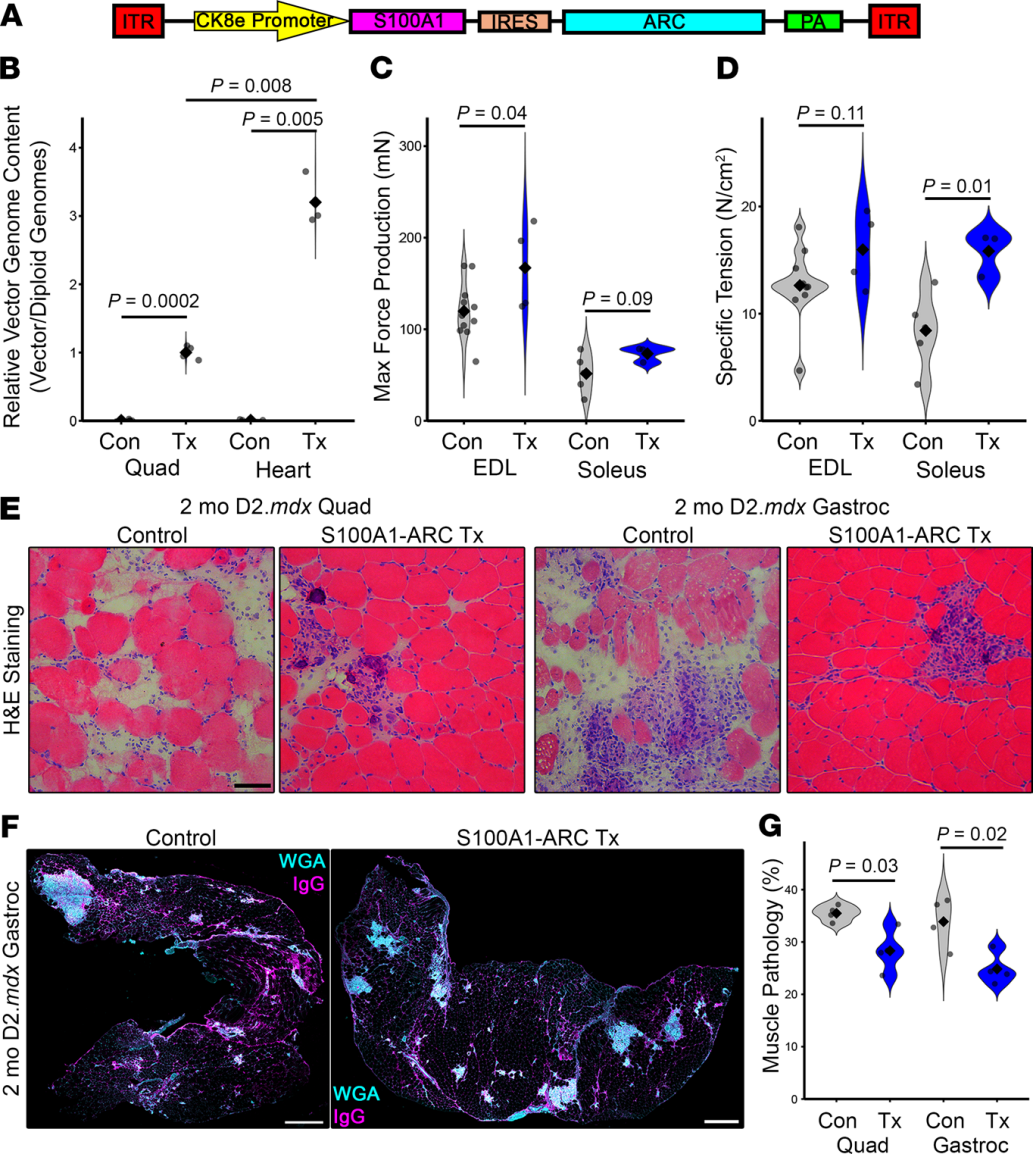
S100A1-ARC benefits D2.*mdx* skeletal muscle. (**A**) Male D2.*mdx* mice received treatment with control (Con; *n* = 11) or S100A1-ARC driven by the striated muscle–specific CK8e promoter (Tx; *n* = 4) at 1 month of age and underwent ex vivo functional measures of the soleus and extensor digitorum longus (EDL) muscles at 2 months. (**B**) Vector genomes were quantified in the quadriceps and hearts from Con and Tx mice. (**C**) Maximum tetanic force production and (**D**) specific tension were performed on the EDL and soleus muscles of Con and Tx D2.*mdx* mice at study endpoint. (**E**) Representative H&E staining of quadriceps (Quad) and gastrocnemius (Gastroc) muscles from Con and Tx D2.*mdx* are shown at study endpoint (scale bar: 100 μm). (**F**) Representative wheat germ agglutinin (WGA) and endogenous mouse IgG staining of Con and Tx Gastroc (scale bar: 500 μm) and (**G**) accompanying quantification of total muscle pathology are shown for Quad and Gastroc muscle sections from this study. Data are displayed as violin plots with individual values indicated by circles and were analyzed using (**B**) 2-factor ANOVA (treatment and muscle effects) followed by multiple *t* tests with Bonferroni’s correction (α = 0.05; *P < 0.05 vs. muscle-matched treatment values; #*P* < 0.05 vs. treatment-matched Quad values) and (**C**, **D**, and **G**) 2-tailed Welch’s *t* test (α = 0.05; *P < 0.05 vs. Con values).

Given the protective effect of S100A1-ARC in the dystrophic heart, we sought to determine whether dystrophic skeletal muscle can also benefit from treatment with this bicistronic transgene, including the reduction in severity of muscle degeneration that occurs early in life for D2.*mdx* mice (44). We investigated systemic treatment of S100A1-ARC driven by the CK8e striated muscle promoter (64) (Figure 7A) delivered at 1 month, with an endpoint at 2 months to gauge potential protection at the peak of D2.*mdx* skeletal myopathy. At the endpoint, the quadriceps of treated mice exhibited robust levels of vector transduction (Figure 7B); however, this transduction was lower than that found in the heart, similar to previous reports (16, 65). Importantly, treated extensor digitorum longus muscles exhibited greater maximum force production than controls, whereas the soleus muscles of treated mice demonstrated significantly higher specific tension (tetanic force normalized to muscle cross-sectional area; Figure 7, C and D). In agreement with the improved muscle force production in the extensor digitorum longus and soleus, S100A1-ARC–treated quadriceps and gastrocnemius muscles showed less histopathology than control muscles, as observed with H&E staining (Figure 7E). To quantify aspects of muscle pathology associated with this early-stage D2.*mdx* disease progression, we stained quadriceps and gastrocnemius sections with fluorescently tagged wheat germ agglutinin (WGA), which delineates muscle fibers from the interstitium, and anti-mouse IgG, which marks serum protein leakage into damaged muscle. As shown in Supplemental Figure 9A, this staining combination revealed different forms of muscle pathology, including inflammatory lesions, regenerative foci, myofiber damage/necrosis, and edema, which can be cumulatively quantified as total muscle pathology using the method illustrated in Supplemental Figure 9B. Pathology analysis using this methodology revealed that significant reductions in muscle pathology of the quadriceps and gastrocnemius were achieved as a result of S100A1-ARC treatment (Figure 7, F and G). These data suggest that S100A1-ARC effectively reduces the extent of muscle fiber death and preserves muscle function during early-stage D2.*mdx* skeletal muscle pathology. Therefore, the protective effect of this therapeutic dual transgene strategy is not restricted to the myocardium and may represent an impactful therapy for all muscles affected by DMD.

## Discussion

Effective therapeutics for DMD cardiomyopathy are a major unmet clinical need for individuals affected by this devastating disease. Herein, we have identified that AAV-mediated cardiac expression of S100A1 prevents many features of diastolic dysfunction in the dystrophic heart, and cardiac ARC overexpression prolongs the life expectancy of D2.*mdx* mice. The combination of the 2 transgenes in a bicistronic vector improves the functional outcomes and survival of D2.*mdx* hearts and exhibits long-term safety and evidence of cardiac benefits in the GRMD canine model of DMD. Furthermore, S100A1-ARC expression in skeletal muscle reduces pathology and functional decrements associated with the early stage of D2.*mdx* muscle disease. Taken together, these findings indicate that the dual expression of S100A1 and ARC in dystrophic muscle represents a promising gene therapy for the treatment of DMD.

In this report, we rigorously demonstrate that progressive diastolic dysfunction and LV restriction are major features of the cardiomyopathy exhibited by the D2.*mdx* murine model of DMD, characterized by pronounced reductions in EDV, SV, and MV E/A. This phenotype, which entails an impairment in the LV’s ability to relax and fill with blood before the next contraction, is becoming a more recognized aspect of DMD cardiomyopathy that precedes development of systolic dysfunction and DCM (11, 12). Interestingly, this cardiac phenotype closely resembles heart failure with preserved EF, a lethal form of cardiomyopathy that commonly affects individuals with metabolic diseases (66), where reduced EDV and SV result in compensatory increases in heart rate and contractility to maintain adequate cardiac output for survival. Therefore, we suspect this restrictive phase of DMD cardiomyopathy represents a similar silent form of heart failure that can occur for a considerable period before progressive myocardial damage and energy deficits lead to sudden death or decompensation to DCM. Therefore, therapeutics effective at treating this phase of DMD cardiomyopathy have the potential to delay onset of DCM, as evidenced by our previous observations using the phosphodiesterase 5 inhibitor, tadalafil (17, 67).

There is a prevailing hypothesis that DMD cardiomyopathy is largely driven by impaired calcium handling (20–22). Accordingly, we (16) and others (55) have reported resting calcium levels to be elevated in dystrophic cardiomyocytes. These elevated calcium levels can lead to a myriad of negative consequences for the cardiomyocyte, including impaired relaxation, E-C coupling abnormalities, mitochondrial dysfunction, aberrant protease activation, and, ultimately, cell death (17, 20, 23, 24, 68–70). At the organ level, these consequences of cardiomyocyte calcium mishandling result in diastolic dysfunction, life-threatening arrhythmias, and progressive replacement of myocardium with fibrotic scarring, which are all major features of DMD cardiomyopathy (4, 7, 10).

We sought to address these calcium-associated issues of the dystrophic myocardium by implementing gene therapy to upregulate cardiomyocyte S100A1 and ARC, alone and in combination. These proteins are both involved in calcium homeostasis (35, 71), and we identified differential benefits incurred by each transgene: S100A1 alleviates LV restriction and ARC prolongs life expectancy. Physiologically, these distinct effects are logical. S100A1 is an EF-hand calcium sensor that can directly buffer calcium, increase SERCA activity, and reduce ryanodine receptor 2 leakage upon activation by calcium binding (35), thereby reducing cytosolic calcium levels through multiple mechanisms to allow improved myofilament relaxation. ARC can also directly bind calcium (71); however, its primary mechanism of action is to inhibit activation of cell death pathways, including apoptosis (intrinsic and extrinsic pathways), necroptosis, and pyroptosis (72). Because ARC overexpression has previously been shown to decrease cardiomyocyte death in the border zone of postischemic myocardium (36), ARC possibly prevents other forms of cardiomyocyte death, including mechanoptosis (73). Therefore, ARC ultimately reduces loss of cardiomyocytes and, as a result, enhances organism survival.

To take advantage of both major benefits incurred by cardiac overexpression of S100A1 and ARC, a bicistronic DNA construct to deliver both transgenes in the same self-complementary AAV vector was developed. This dual transgene approach resulted in long-term improvements in D2.*mdx* diastolic function and survivability, heart failure prevention when combined with a cardiotoxic μDys transgene, and long-term safety and histological benefits when delivered to the hearts of GRMD canines. These findings indicate that S100A1-ARC gene therapy represents a promising treatment for DMD cardiomyopathy that addresses a current unmet clinical need for individuals with DMD. From a translational perspective, clinical implementation of this cardiac-directed dual transgene vector has high feasibility via the intracoronary delivery we used in canines. This delivery requires considerably less vector than systemic AAV treatments that aim to transduce all musculature (61), therefore improving the overall safety profile of AAV delivery, which has been associated with severe and lethal responses in the clinic after systemic delivery with high doses (74, 75). Furthermore, this strategy to upregulate endogenously expressed genes, rather than delivering a foreign transgene, should prevent incidence of transgene immunity, as has been identified in recent gene therapy recipients (76).

Other gene therapy approaches to address DMD cardiomyocyte calcium handling issues primarily involve increasing SERCA activity. This includes directly overexpressing SERCA (26) and manipulating levels of SERCA-modulating proteins (27, 28). Although these approaches do effectively increase SERCA activity, it is unclear how the increased energy requirements of additional active calcium transport affect energetically challenged dystrophic cardiomyocytes (20). Furthermore, they do not address the release of calcium from the sarcoplasmic reticulum through leaky ryanodine receptor channels. S100A1 overexpression, on the other hand, effectively addresses these calcium handling issues while also providing direct mitochondrial benefits to increase ATP production in cardiomyocytes (77). The pleiotropic benefits of S100A1 in cardiomyocyte calcium handling have led to efforts to develop S100A1-based gene therapies for several forms of heart failure (35), including a recent report that utilized AAV5-packaged S100A1 to improve functional outcomes in a porcine model of myocardial infarction (78). To our knowledge, the current work is the first to exploit S100A1’s myriad benefits in the context of DMD cardiomyopathy, which showed promising efficacy at preventing diastolic dysfunction and LV restriction in the severe D2.*mdx* mouse model. Particularly in combination with the life-extending benefits of ARC, we anticipate the S100A1-ARC strategy developed in this study to be effective at improving cardiac outcomes in other muscular dystrophies, such as the limb-girdle muscular dystrophies, as well as other forms of cardiomyopathy/heart failure, including hypertrophic cardiomyopathy and DCM caused by various genetic and environmental factors.

In addition to the robust cardiac benefits achieved by S100A1-ARC gene therapy, we also provide evidence that this dual-transgene vector improves skeletal muscle phenotypes when driven by the striated muscle–specific CK8e promoter (64). The severe degenerative phase of D2.*mdx* skeletal myopathy peaks at approximately 2 months (44); therefore, the improved muscle function and histopathology observed in this study after S100A1-ARC treatment was incurred during the most devastating stage of murine muscle disease progression. This was achieved without sarcolemma-stabilizing treatments, like μDys expression. Although additional studies are required to determine optimal dosing and timing of S100A1-ARC as a standalone or adjunct gene therapy for skeletal muscle, these data provide compelling proof-of-concept that S100A1-ARC is protective in the early phases of D2.*mdx* skeletal muscle pathology and represents a possible treatment for all striated muscles affected by DMD.

The past decade has seen much progress in the field of DMD therapeutics with the regulatory approval of several treatments, including small molecules (79–81), antisense oligonucleotides (82), and AAV gene therapy (62). Despite these advancements in therapeutics focused on skeletal muscle outcomes, DMD cardiomyopathy is still primarily managed by treatment with beta-blockers, ACE inhibitors/angiotensin receptor blockers, and/or mineralocorticoid receptor agonists (83, 84). In this report, we identify S100A1-ARC gene therapy as an effective treatment for DMD cardiomyopathy through rigorous efficacy testing in D2.*mdx* mice and a safety evaluation in the GRMD large animal model of DMD. This discovery is very timely, as systemic μDys gene therapy is now approved for DMD (62), and it is currently unknown how these miniaturized dystrophin transgenes modify the progression of DMD cardiomyopathy in the clinic. The fact that high cardiac expression of some μDys transgenes can induce premature heart failure and death in mice is very concerning (16); however, it is also unclear what expression levels are being experienced in the human heart after gene therapy. Nonetheless, our findings indicate that S100A1-ARC therapy represents a promising treatment strategy for DMD cardiomyopathy that can be administered alone or in combination with μDys and, likely, other emerging dystrophin-replacement strategies. Furthermore, the dystrophin-independent nature of this therapeutic approach conveys a high probability of also being advantageous for the treatment of other muscular dystrophies, myopathies, and cardiomyopathies where myocyte calcium dysregulation and death are driving mechanisms.

## Methods

### Sex as a biological variable.

This study only involved the use of male animals because DMD is an X-linked disease that primarily affects human males.

### Animals.

This study used male WT (D2.WT; Jax strain 000671) and *mdx* (D2.*mdx*; Jax strain 013141) mice on the DBA/2J genetic background from colonies originally obtained from The Jackson Laboratory (43, 44). Mice were housed 1–5 per cage; randomly assigned into groups; provided ad libitum access to food (NIH-31 open formulation diet; Envigo, 7917), water, and enrichment; and maintained on a 12-hour light/12-hour dark system. GRMD canines from our established colony were housed and cared for as previously described (17, 85). Mouse treatments entailed i.v. delivery of vector via tail-vein injections, as previously described (16). Mavacamten/MYK-461 (Selleckchem, S8861) was diluted in cherry syrup vehicle (Humco, 00395266216) and administered per oral at a dose of 2.5 mg/kg.

### Canine intracoronary vector delivery.

Metoclopramide (0.5 mg/kg) and maropitant citrate (1 mg/kg) were s.c. administered prior to the procedure and an i.v. catheter was placed routinely. Dogs were induced with propofol (2–10 mg/kg, i.v.) and intubated, and anesthesia was maintained with isoflurane (1%–3%) with additional propofol and fentanyl (0.7 mcg/kg/min) as necessary. After induction, dogs were placed in right lateral recumbency and the right groin region was clipped of fur and aseptically prepared in a routine manner. A skin incision was performed over the right femoral artery using a scalpel blade. A 6 Fr introducer was placed using the modified Seldinger technique. A 5 French pigtail catheter was inserted through the introducer and advanced into the aortic root. A contrast study was performed with 1 mL/kg of iodinated contrast using a power injector to highlight coronary arteries. Various coronary catheters were selected based on the coronary anatomy observed on the angiogram (4 French diagnostic coronary catheters). Small volumes of diluted contrast were injected during the procedure to ensure good seating of the coronary catheter in the coronary ostium. A total dose of 1 × 10^13^ to 5 × 10^13^ gc of vector (up to 5 mL volume) was administered, with two-thirds of the volume administered into the left coronary and one-third of the total volume administered into the right coronary. Before vector administration into each coronary artery, a CRI of adenosine was initiated at a dose of 1 mg/kg/min. The vector was then injected over 15 seconds and the adenosine infusion was continued for an additional 30 seconds. After administration in both coronary arteries, the catheter was removed. The femoral artery was ligated proximal to the catheter insertion site with 2-0 silk circumferential sutures and 1 circumferential suture placed distally. The incision was closed in 3 layers (muscle, subcutaneous, and intradermal) in a simple continuous pattern using 3-0 polydioxanone.

### AAV vector production.

Codon-optimized murine S100A1 (*S100a1*) and ARC (*Nol3*) open reading frames were synthesized by GenScript and cloned into a pAAV shuttle plasmid containing the cardiac-specific cTnT promoter (57) and a minimized synthetic polyadenylation signal sequence (86). The bicistronic construct contained native canine *S100A1*, an internal ribosomal entry site (60), and codon-optimized canine ARC (*HSF4*) sequences, and was driven) by either the cTnT or CK8e (64) promoters. AAV vector packaging was performed using the triple-transfection method, as previously described (59, 87).

### Echocardiography and ECG.

Mouse ECGs and transthoracic echocardiograms were performed using the Vevo 3100 preclinical imaging system (Fujifilm VisualSonics), as previously described (16, 88), by investigators blinded to experimental design. Mice were anesthetized using 3% isoflurane and maintained at 1.5%–2% to keep heart and respiration rates consistent among treatment groups. Body temperature was maintained at 37**°**C throughout imaging. ECGs were imported into LabChart (ADInstruments) for analysis. Four images were acquired for each animal: B-mode parasternal long axis, B-mode short axis, M-mode short axis, and apical 4-chamber view with color Doppler and pulsed-wave Doppler. M-mode short-axis images were acquired at the level of the papillary muscle. Flow through the MV was sampled at the point of highest velocity, as indicated by aliasing, with the pulsed-wave angle matching the direction of flow. Images were imported into Vevo LAB for analysis. Measurements of M-mode short-axis and pulsed-wave Doppler images were made from 3 consecutive cardiac cycles between respirations. Echocardiography measurements in canines were performed as described previously (17), using a Philips CX-50 system and an 8- to 3-MHz transducer.

### Ex vivo muscle function.

Maximum tetanic tension of the extensor digitorum longus and soleus muscles was evaluated as previously described (43, 44, 85) by experimentally blinded members of the University of Florida Physiological Assessment Core. After experimental procedures, muscles were weighed, frozen in OCT or snap-frozen, and stored at –80°C until further use.

### Immunoblotting.

Immunoblotting was performed as previously described (43, 45) using the following primary antibodies: anti-S100A1 (Abcam, 11428), anti-ARC/NOL3 (Proteintech, 10846-2-AP), anti-dystrophin [DSHB, MANEX1011B(1C7)], and anti-GFP (Abcam,13970). Enhanced chemiluminescent signals were acquired and quantified using the Licor C-Digit system, with signal intensity normalized to sample protein loading, as visualized using Ponceau red staining.

### Gene expression and vector genome quantification.

Gene expression analysis was conducted as previously described (43, 45) using the following mouse-specific primers: *Nppb* (fwd) 5′-TTTGGGCTGTAACGCACTGA-3′ and (rev) 5′-CACTTCAAAGGTGGTCCCAGA-3′; *Myh6* (fwd) 5′-AACCTGTCCAAGTTCCGCA-3′ and (rev) 5′-ATTCCTCGTCGTGCATCTTCTTG-3′; *Myh7* (fwd) 5′-TTGCTACCCTCAGGTGGCT-3′ and (rev) 5′-CCTTCTCAGACTTCCGCAGG-3′; *Rpl32* (fwd) 5′-GGGTGCGGAGAAGGTTCAA-3′ and (rev) 5′-GCAATCTCAGCACAGTAAGATTTGT-3′. Relative gene expression quantification was performed using the ΔΔCt method with *Rpl32* as the normalization gene.

Vector genome content quantification was performed as previously described (16). Primers used during this assay include those for AAV2 ITR (recognizes vector genomes; fwd: 5′-GGAACCCCTAGTGATGGAGTT-3′; rev: 5′-CGGCCTCAGTGAGCGA-3′) and a genomic DNA region in the *Rpl32* locus of murine chromosome 6 (recognizes diploid genomes; fwd: 5′-GAGAAGGTTCAAGGGCCAGAT-3′; rev: 5′-AGCTCCTTGACATTGTGGACC-3′). Canine diploid genomes were quantified using primers specific for the genomic DNA regions in the *FUCA1* locus of canine chromosome 2 (fwd: 5′-CGCGCTTCTTCCACCCCGACACCT-3′; rev: 5′-CCCCCGCCCCGAGCAGACG-3′). Vector genome expression was quantified and normalized to diploid genome expression using the ΔΔCT method.

### Histology.

H&E and Picrosirius red staining were performed as previously described (43, 44). Slides were visualized with a Leica DMR microscope, and images were acquired using a Leica DFC310FX camera interfaced with Leica LAS X software. Images were processed and analyzed by investigators blinded to study groups using ImageJ (NIH) software. Comparative Picrosirius red quantification was performed using slides that were stained and imaged together. Immunofluorescence was performed as previously described (16, 43) using anti-dystrophin [DSHB, MANHINGE4A(5C11)] and anti-ARC/NOL3 (Proteintech,10846-2-AP) primary antibodies. Alexa Fluor 488–conjugated WGA (Invitrogen, W11261) and Alexa Fluor 568–conjugated goat anti-mouse IgG (Invitrogen, A-11004) were used to delineate muscle fibers and identify damaged muscle fibers, respectively. All fluorescent imaging slides were mounted with Prolong Gold mounting media (Invitrogen, P36934), and image acquisition was performed using a STELLARIS 5 white-light laser confocal system (Leica Microsystems).

### Statistics.

Statistical analysis was performed using an unpaired, 2-tailed Welch’s *t* test (α = 0.05), proper form of ANOVA (1-factor, 2-factor, or repeated measures) followed by post hoc tests (α = 0.05) and Kaplan-Meier estimator analyses (α = 0.05), where appropriate. A *P* value less than 0.05 was considered significant. Data are displayed as violin plots with individual values or survival curves, unless otherwise indicated.

### Study approval.

All studies were approved by and conducted in accordance with the University of Florida IACUC.

### Data availability.

The datasets generated in the current study are available in the Supporting Data Values file.

## Author contributions

DWH and HLS conceptualized the study and obtained funding. DWH, CCH, EZ, KIL, YL, and MMS conducted experiments and acquired data. DWH, CCH, EAZ, MMS, and HLS analyzed data, interpreted data, and participated in manuscript writing.

## Conflict of interest

HLS and MMS are inventors on patent US20210260215A1. HLS and DWH are inventors on patent application 63/869,080.

## Funding support

This work is the result of NIH funding, in whole or in part, and is subject to the NIH Public Access Policy. Through acceptance of this federal funding, the NIH has been given a right to make the work publicly available in PubMed Central.

- NIH National Institute of Arthritis and Musculoskeletal and Skin Diseases P50-AR052646 (to HLS and DWH) and R01-AR083108 (to DWH).
- NIH National Institute of Child Health and Development P50-HD119693 (to HLS and DWH).
- Parent Project Muscular Dystrophy (to HLS).
- Leducq Foundation 13CVD04 (to HLS).
- US Department of Defense W81XWH-22-1-1070 (to DWH).

## Supplementary Material

Supplemental data

Unedited blot and gel images

Supporting data values

## Version 1 06/09/2026

In-Press Preview

## Version 2 08/10/2026

Electronic publication
