# Supplementary material for: Combination S100A1 and ARC gene therapy as a treatment for Duchenne muscular dystrophy cardiomyopathy
Source: JCI Insight. 2026 Jun 9;11(15):e204852. doi: 10.1172/jci.insight.204852 (PMC13464013; doi:10.1172/jci.insight.204852)
Supplement: Supplementary file 1 [file jciinsight-11-204852-s249.pdf]

SUPPLEMENTAL MATERIALS

SUPPLEMENTAL TABLES

Supplemental Table 1. Electrocardiogram measures from D2.WT and D2.*mdx* mice

Age (mo)	Genotype	RR Interval (ms)	Heart Rate (BPM)	PR Interval (ms)	P Duration (ms)
2	WT	146.68±5.64	411.36±14.49	39.76±1.26	7.95±0.35
	mdx	143.10±5.01	422.98±13.22	37.61±1.63	8.04±0.21
3	WT	132.73±5.44	455.45±16.55	34.46±0.86	8.19±0.97
	mdx	139.15±4.09	433.10±13.18	33.96±0.78	7.60±0.33
4	WT	134.84±2.80	445.76±9.52	34.76±1.36	6.94±0.22
	mdx	136.85±2.14	439.44±6.50	35.07±0.95	7.87±0.27
6	WT	139.19±4.01	435.36±11.58	36.12±0.95	7.51±0.37
	mdx	151.59±5.58	400.44±14.06	35.84±0.85	8.01±0.71
8	WT	135.09±3.77	446.34±11.28	34.89±0.99	7.75±0.40
	mdx	141.03±2.23	426.25±6.68	34.43±1.18	8.24±0.72
10	WT	138.78±3.94	435.30±11.61	34.36±1.12	7.84±0.34
	mdx	141.00±1.85	426.01±5.71	35.17±1.10	7.79±0.46
12	WT	139.30±3.99	432.77±11.83	33.10±1.13	8.06±0.36
	mdx	140.84±3.31	427.63±10.08	34.84±0.62	7.59±0.63
ANOVA P-values	Overall	0.21	0.13	0.06	0.98
	Genotype	0.59	0.53	0.23	0.91
	Age	0.12	0.14	***0.0033***	0.94
	Genotype*Age	0.58	0.47	0.79	0.78

Data are presented as mean±SEM and were analyzed using two-factor ANOVA (genotype and age effects) followed by multiple T-tests with Bonferonni correction (α = 0.05). *p < 0.05 vs. age-matched WT values.

Supplemental Table 1 Continued

Age (mo)	Genotype	QRS Interval (ms)	QT Interval (ms)	QTc Interval (ms)	JT Interval (ms)
2	WT	13.47±1.53	17.56±1.61	45.81±3.94	4.09±0.85
	mdx	11.29±0.37	20.89±0.77*	55.36±2.06*	9.61±0.81*
3	WT	9.84±0.41	16.18±0.62	44.52±1.82	6.34±0.84
	mdx	10.32±0.30	17.84±0.35	47.93±1.38	7.52±0.23
4	WT	10.47±0.89	15.91±0.53	43.37±1.56	5.44±0.48
	mdx	10.74±0.22	19.17±0.52*	51.83±1.28*	8.43±0.49*
6	WT	10.37±0.37	16.63±0.47	44.77±1.44	6.27±0.60
	mdx	10.30±0.50	19.51±0.46*	50.18±0.74*	9.21±0.51*
8	WT	10.48±0.74	16.12±0.77	44.07±2.40	5.64±0.57
	mdx	10.13±0.47	19.12±0.44*	50.99±1.39*	8.99±0.47*
10	WT	10.25±0.71	18.00±0.66	48.46±1.96	7.75±0.54
	mdx	10.43±0.67	19.78±0.44	52.66±1.04	9.34±0.54
12	WT	9.85±0.92	16.95±0.47	45.43±1.01	7.09±0.98
	mdx	10.24±0.44	20.82±0.68*	55.61±2.12*	10.59±0.65*
ANOVA P-values	Overall	0.10	***<0.0001***	***<0.0001***	***<0.0001***
	Genotype	***0.023***	***0.0014***	***0.0010***	***<0.0001***
	Age	***0.0090***	***0.013***	0.080	***0.013***
	Genotype*Age	0.44	0.63	0.44	0.077

Data are presented as mean±SEM and were analyzed using two-factor ANOVA (genotype and age effects) followed by multiple T-tests with Bonferonni correction (α = 0.05). *p < 0.05 vs. age-matched WT values.

Supplemental Table 1 Continued

Age (mo)	Genotype	T_{peak} T_{end} Interval (ms)	P Amplitude (V)	Q Amplitude (V)
2	WT	2.63±0.62	108.04±14.35	4.19±3.11
	mdx	6.68±0.69*	176.46±14.81*	18.96±3.05
3	WT	3.83±0.49	127.32±10.47	13.46±3.72
	mdx	4.76±0.22	148.53±15.68	11.64±2.72
4	WT	3.50±0.36	112.89±11.76	14.45±3.76
	mdx	5.74±0.44*	149.40±6.52*	16.51±3.53
6	WT	3.96±0.40	117.02±10.37	13.23±3.50
	mdx	6.31±0.76*	104.16±8.59	16.31±2.47
8	WT	3.97±0.58	103.24±9.71	6.53±3.60
	mdx	6.49±0.59*	98.97±14.72	17.38±7.30
10	WT	5.60±0.59	104.22±7.37	16.08±5.02
	mdx	6.15±0.64	98.56±9.42	12.66±12.15
12	WT	4.72±0.65	112.73±4.97	15.42±2.84
	mdx	7.06±0.63*	103.85±13.64	5.30±8.18
ANOVA P-values	Overall	***<0.0001***	***<0.0001***	0.75
	Genotype	***<0.0001***	***0.0002***	0.078
	Age	0.084	***0.0006***	0.96
	Genotype*Age	0.15	***0.0056***	0.33

Data are presented as mean±SEM and were analyzed using two-factor ANOVA (genotype and age effects) followed by multiple T-tests with Bonferonni correction (α = 0.05). *p < 0.05 vs. age-matched WT values.

Supplemental Table 1 Continued

Age (mo)	Genotype	R Amplitude (V)	S Amplitude (V)	T Amplitude (V)
2	WT	526.58±82.98	-271.39±66.80	97.33±24.77
	mdx	640.01±31.32	-434.63±62.74	316.12±36.68*
3	WT	574.45±49.26	-276.58±81.13	217.69±53.93
	mdx	516.27±41.67	-381.04±84.66	298.87±23.57
4	WT	598.22±103.36	-262.56±135.35	235.32±40.83
	mdx	648.39±60.52	-478.20±89.14	316.32±33.01
6	WT	510.52±39.85	-245.54±53.24	214.28±26.84
	mdx	566.08±50.45	-412.47±121.80	318.25±35.20*
8	WT	526.98±50.96	-143.59±64.31	218.69±38.14
	mdx	495.33±49.27	-96.30±73.77	291.89±33.75
10	WT	487.88±63.63	-115.00±59.98	276.64±27.12
	mdx	506.91±52.09	-386.66±126.10*	401.71±48.10*
12	WT	496.76±42.21	-61.00±47.21	228.85±46.46
	mdx	554.03±36.20	-551.79±73.68*	398.49±36.33*
ANOVA P-values	Overall	0.4	***0.0002***	***<0.0001***
	Genotype	0.19	0.23	***0.0003***
	Age	0.33	0.077	***0.028***
	Genotype*Age	0.82	0.11	0.47

Data are presented as mean±SEM and were analyzed using two-factor ANOVA (genotype and age effects) followed by multiple T-tests with Bonferonni correction (α = 0.05). *p < 0.05 vs. age-matched WT values.

Supplemental Table 2. Heart and cardiomyocyte size in 12 mo D2.WT and D2.*mdx* mice

Genotype	WT	*mdx*	P-value
Heart Mass (mg)	196.96±3.96	146.24±5.38	0.0000000052
Heart Mass:TL (mg/mm)	10.81±0.40	8.26±0.28	0.0000053
LV Length (mm)	8.34±0.07	7.90±0.16	0.019
Cardiomyocyte MFD (μm)	24.36±1.19	17.23±1.86	0.022

Data are presented as mean±SEM and were analyzed using two-tailed Welch's T-test (α = 0.05)
TL- tibia length
LV- Left ventricle
MFD- minimum Feret diameter

Supplemental Table 3. LV Restriction in Pre-DCM GRMD canines

Genotype	Normal	Carrier	GRMD
**Sample size (n)**	15	42	31
**Age (mo)**	17.13±2.30	20.93±1.07	17.06±1.19
**LVEDD (cm)**	3.90±0.08	3.97±0.07	3.47±0.09*#
**LVESD (cm)**	2.65±0.11	2.79±0.07	2.46±0.10*
**FS (%)**	32.78±1.94	29.69±1.22	29.78±1.46
**LVEDV (mL)**	66.53±3.24	69.92±2.83	51.57±3.35*#
**LVESV (mL)**	26.87±2.68	30.55±1.67	23.51±2.43*
**EF (%)**	60.47±2.49	56.50±1.75	56.88±2.28
**SV (mL)**	39.65±1.85	39.37±2.06	28.06±1.58*#
**MV E (mL/s)**	87.14±5.13	81.11±2.68	73.34±2.19*
**MV A (mL/s)**	55.14±4.05	52.2±2.41	58.33±1.88
**MV E/A**	1.66±0.12	1.68±0.09	1.30±0.04*#

Data are presented as mean±SEM and were analyzed using one-factor ANOVA followed by Tukey post-hoc tests ($\alpha = 0.05$).
LVEDD- left ventricular end diastolic diameter
LVESD- left ventricular end systolic diameter
FS- fractional shortening
LVEDV- left ventricular end diastolic volume
LVESV- left ventricular end systolic volume
EF- ejection fraction
SV- stroke volume
MV E- mitral valve early flow rate
MV A- mitral valve active flow rate
*$p < 0.05$ vs. Normal values
#$p < 0.05$ vs. Carrier values

Supplemental Table 4. GRMD gene therapy summary

Subject	Age of Tx (mo)	Age of Death (yrs)	Endpoint Criteria
GRMD GT1	5	3.9	GI torsion*
GRMD GT2	10	2.9	Pneumonia
GRMD GT3	24	5.5	End of study

*post-mortem specimens were unable to be collected

SUPPLEMENTAL FIGURES

A Cross-Species Alignment of LTBP4 PPR Hinge

```
C57 Mouse    GFLPTRRPEPRPDPGPQPEPRPRPEPR PRPESRPRPEPRPRPEPRPQPESQPRPESRPRPESQPWPEFPLPSIPAWTGPEIPESG
DBA/2J Mouse GFLPTRRPEPRPDPGPQPEPRPRPEPR PRPESRPRPEPRPRPEPRPQPESQP-----------------------WPEFPLPSIPAWTGPEIPESG
Rat          GFLPTRRPEPRPDPGPQPELRPRPEPQ PRPE---------------------------------------------------------------------FPLPSIPAWTGPEIPESG
Pig          GFLPTHRPEPRPEPRP ELRPEPRPEPR PRPE---------------------------------------------------------------------LPLPSIPAWTGPEIPESG
Dog          GFLPTHR---------------------PEPPPEPR PGPE----------------------------------------------------------------------LPLPSIPAWSGPEISESG
Human        GFLPTHRLEPR---------------PEPRPDPRPGPE-----------------------------------------------------------------------LPLPSIPAWTGPEIPESG
```

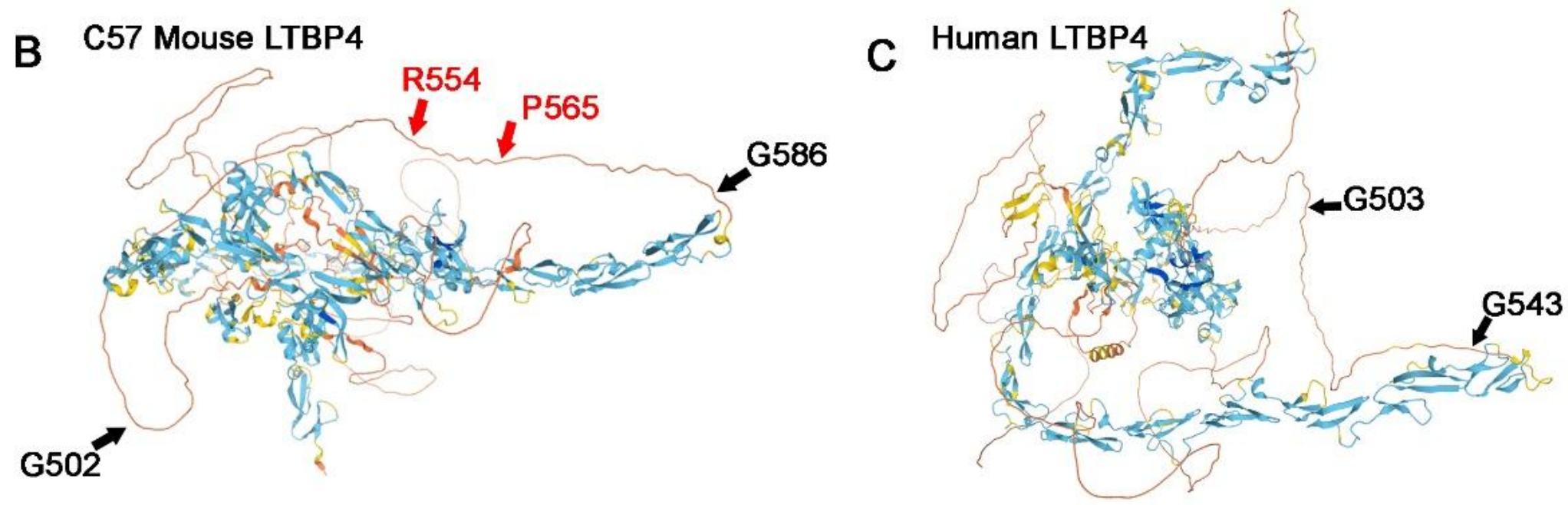

Supplemental Figure 1. Interspecies comparison of LTBP4 PPR hinges. (**A**) Amino acid sequence alignments of the latent TGFβ binding protein 4 (LTBP4) polyproline rich (PPR) hinges of C57 mice, DBA/2J mice, rats, pigs, dogs, and humans. Residues in red correspond to the additional PPR segment found in mice of C57 genetic backgrounds. Alpha-fold generated protein structures of (**B**) C57 mouse and (**C**) human LTBP4 show the PPR hinge boundaries (denoted with black arrows) and the segment found in C57 mice (denoted with red arrows).

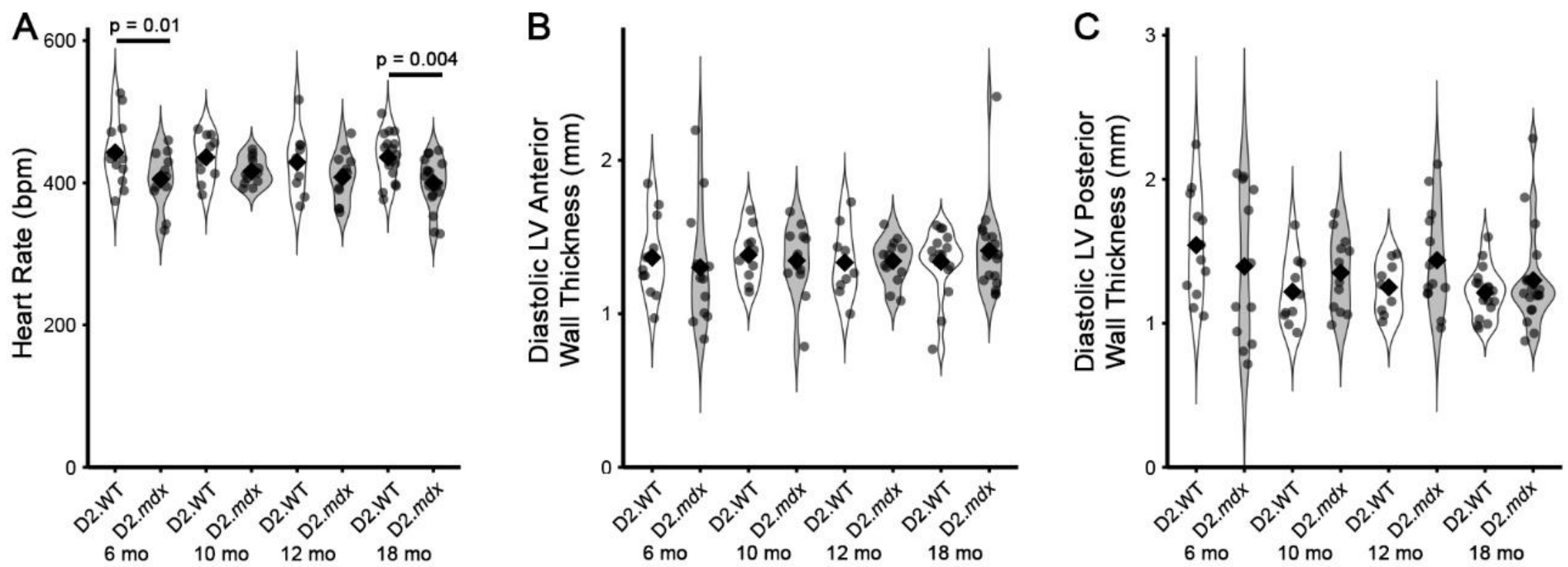

Supplemental Figure 2. Echocardiography measures of D2.WT and D2.*mdx* mice. (**A**) Heart rate, (**B**) diastolic LV anterior wall thickness, and (**C**) diastolic LV posterior wall thickness are shown for 6, 10, 12, and 18 month-old D2.WT and D2.*mdx* mice Data are displayed as violin plots (individual values indicated by circles; means indicated by black diamonds) and were analyzed using two-factor ANOVA (age and genotype effects) followed by multiple T-tests with Bonferroni correction ($\alpha = 0.05$; significant p values are indicated).

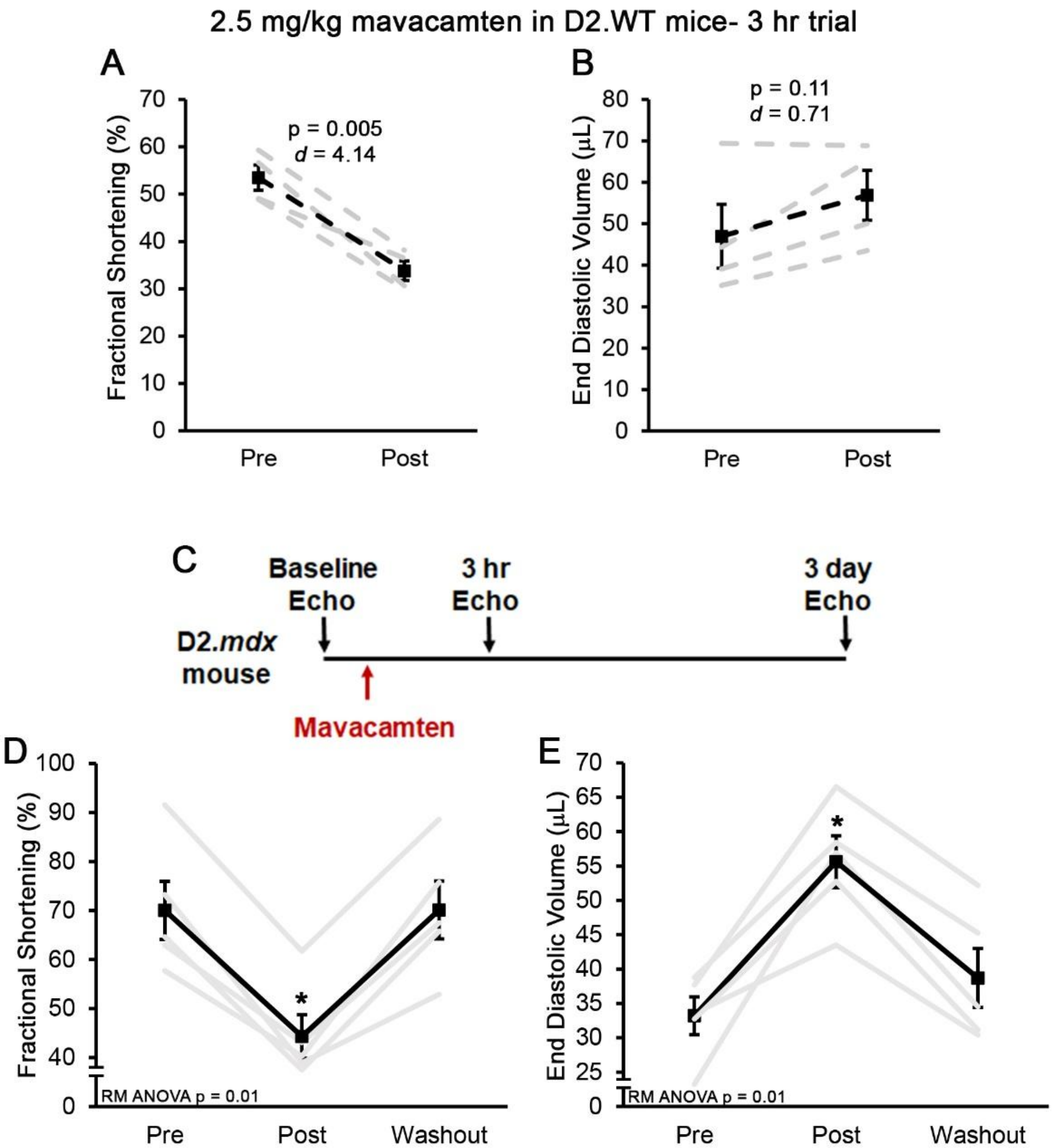

Supplemental Figure 3. Mavacamten acutely reverses left ventricular restriction in D2.*mdx* mice. Male 10 month-old D2.WT mice (n = 4) underwent pre- and post-treatment echocardiography measures to evaluate acute impact of 2.5 mg/kg mavacamten on the contractility of wild-type hearts, as measured by (**A**) fractional shortening, and impact on left ventricular (LV) filling, as shown by (**B**) end diastolic volume. (**C**) Ten month-old male D2.*mdx* mice (n = 5) were subjected to the same imaging and dosing regimen, followed by a 3 day washout measurement. (**D**) Fractional shortening and (**E**) end diastolic volume from this experiment are depicted as mean±SEM black line; dashed for D2.WT; solid for D2.*mdx*) with individual trajectories (gray lines; dashed for D2.WT; solid for D2.*mdx*). Data were analyzed using (**A-B**) two-tailed Welch's T-test (α = 0.05; effect size reported as Cohen's *d*) and (**D-E**) repeated measures (RM) ANOVA followed by Tukey post-hoc tests (α = 0.05; *p <0.05 vs. pre-treatment values).

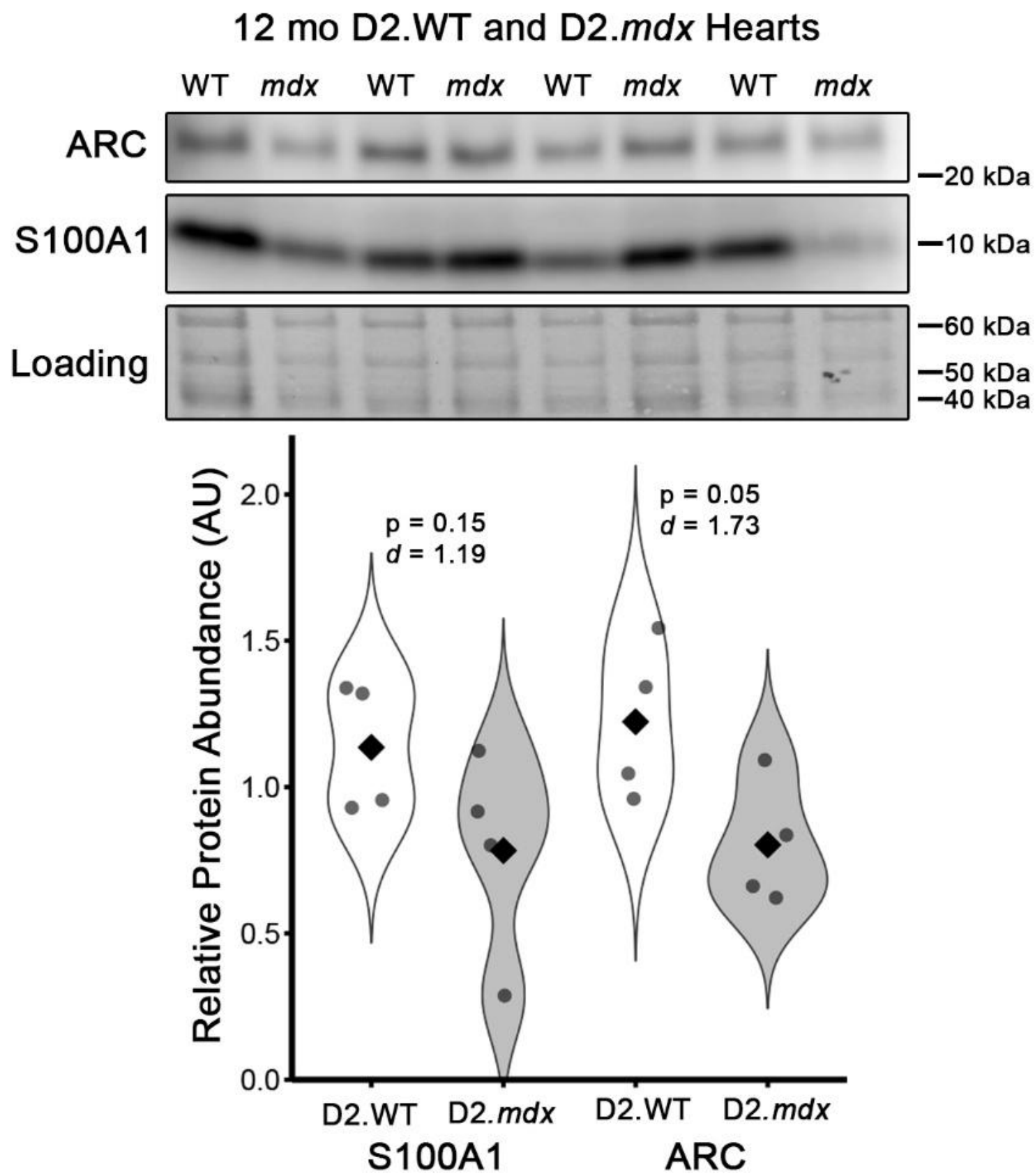

Supplemental Figure 4. S100A1 and ARC levels in D2.WT and D2.*mdx* hearts. Immunoblotting data for S100A1 and ARC in 12 month-old (mo) D2.WT (WT) and D2.*mdx* (*mdx*) hearts (n = 4). Data are displayed as violin plots (individual values indicated by circles; means indicated by black diamonds) and were analyzed using two-tailed Welch's T-test (α = 0.05) with effect size indicated by Cohen's *d* (*d*).

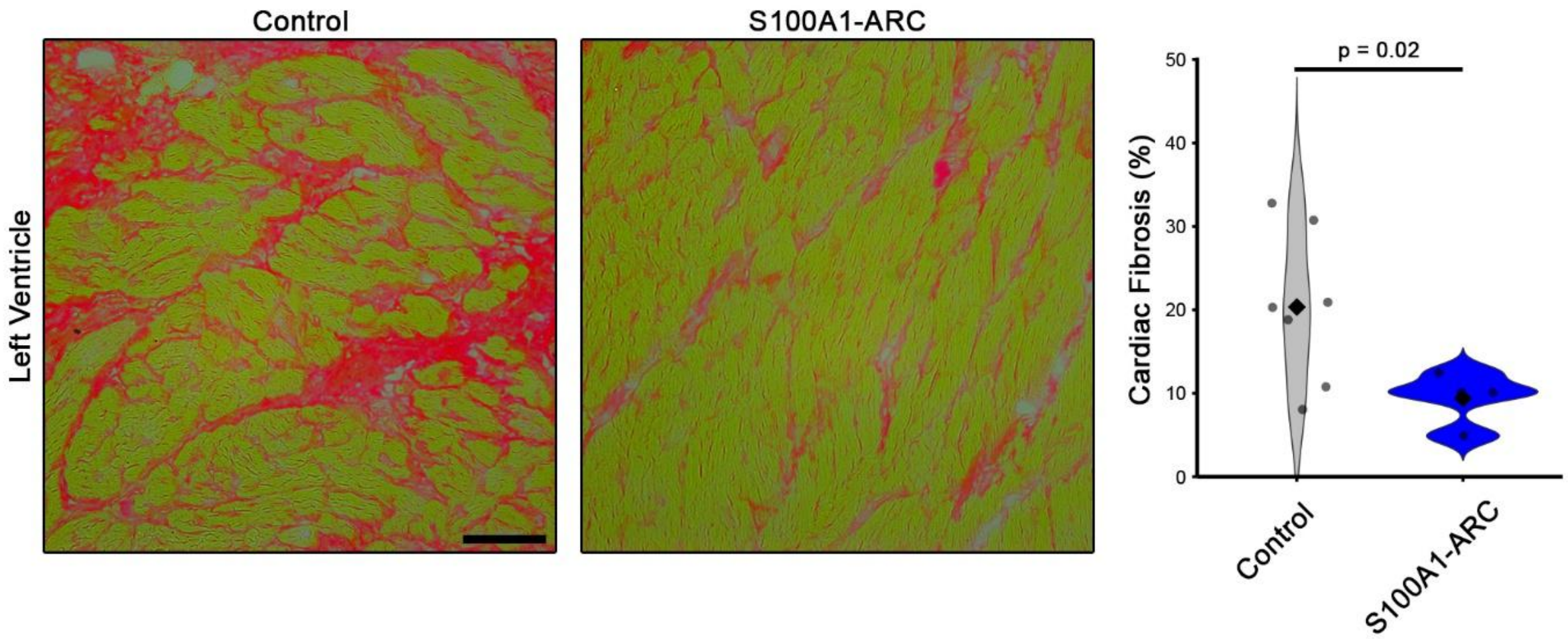

Supplemental Figure 5. S100A1-ARC treatment reduces D2.*mdx* cardiac fibrosis. Representative picrosirius red images and fibrosis quantifications from control (n = 7) and scAAVrh10. cTnT.S100A1-ARC (n = 4) treated 20 mo D2.*mdx* mice. Data are displayed as violin plots (individual values indicated by circles; means indicated by black diamonds) and were analyzed using two-tailed Welch's T-test (α = 0.05; *p < 0.05 vs. control values).

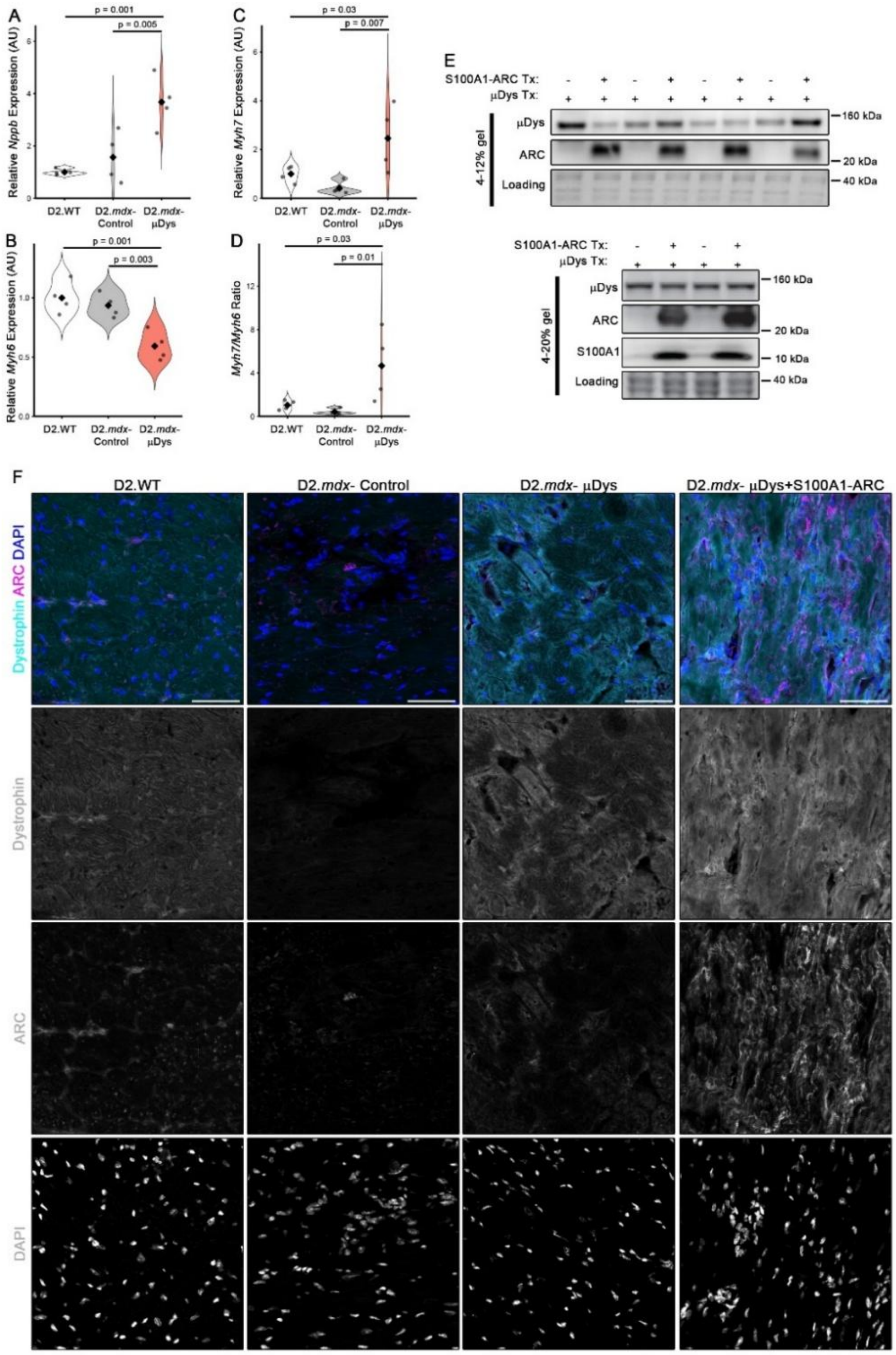

Supplemental Figure 6. Heart failure associated markers and transgene expression in micro-dystrophin treated D2.*mdx* hearts. Gene expression of (**A**) *Nppb*, (**B**) *Myh6*, and (**C**) *Myh7*, as well as (**D**) the *Myh7/Myh6* ratio in 12 month-old D2.WT (n = 4), D2.*mdx* (n = 4), and D2.*mdx* mice that received systemic treatment with the ΔR3-R21ΔCT micro-dystrophin (D2.*mdx*+μDys; n = 4; all reached humane endpoints). (**E**) Immunoblots for μDys, ARC, and S100A1 to confirm expression levels of these transgenes (normalized to Ponceau Red visualized protein loading). (**F**) Immunofluorescent detection of dystrophin/μDys and ARC in heart sections from D2.WT and D2.*mdx* mice that received control, μDys, or μDys+S100A1-ARC treatment (Scale bars = 50 μm). Data are displayed as violin plots (individual values indicated by circles; means indicated by black diamonds) and were analyzed using two-factor ANOVA (age and genotype effects) followed by multiple T-tests with Bonferroni correction (α = 0.05; significant p values are indicated).

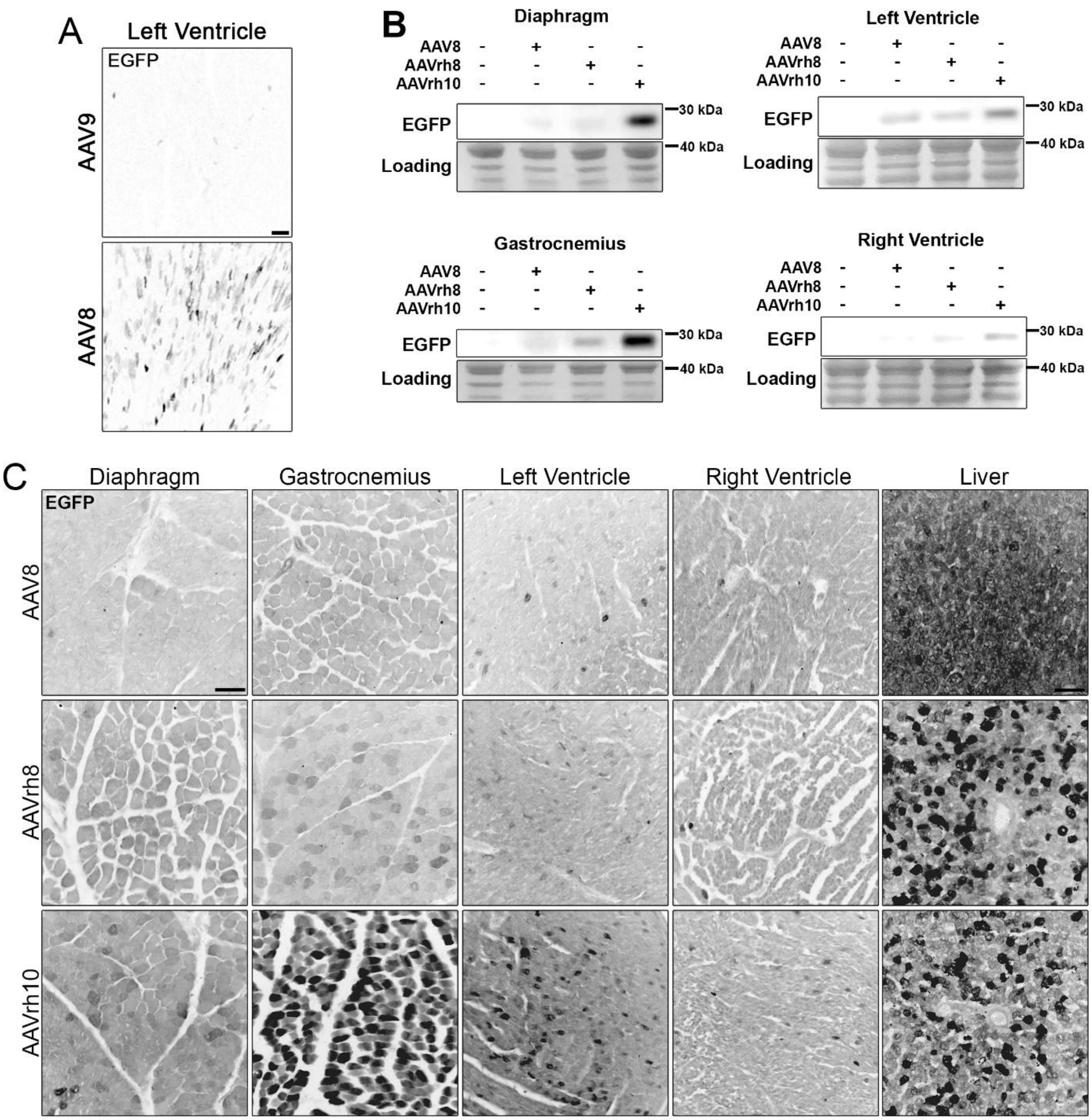

Supplemental Figure 7. Biodistribution of gene therapy vectors in canine striated muscle. (**A**) Representative EGFP fluorescence in the left ventricles of canines 1 week following intravenous administration of AAV8 or AAV9 at a dose of 5x10^{12} gc/kg. (**B**) Immunoblotting and (**C**) representative EGFP fluorescence in canine tissue 1 week following intravenous administration of AAV8, AAVrh8, or AAVrh10 at a dose of 5x10^{12} gc/kg. Scale bars represent 100 μm.

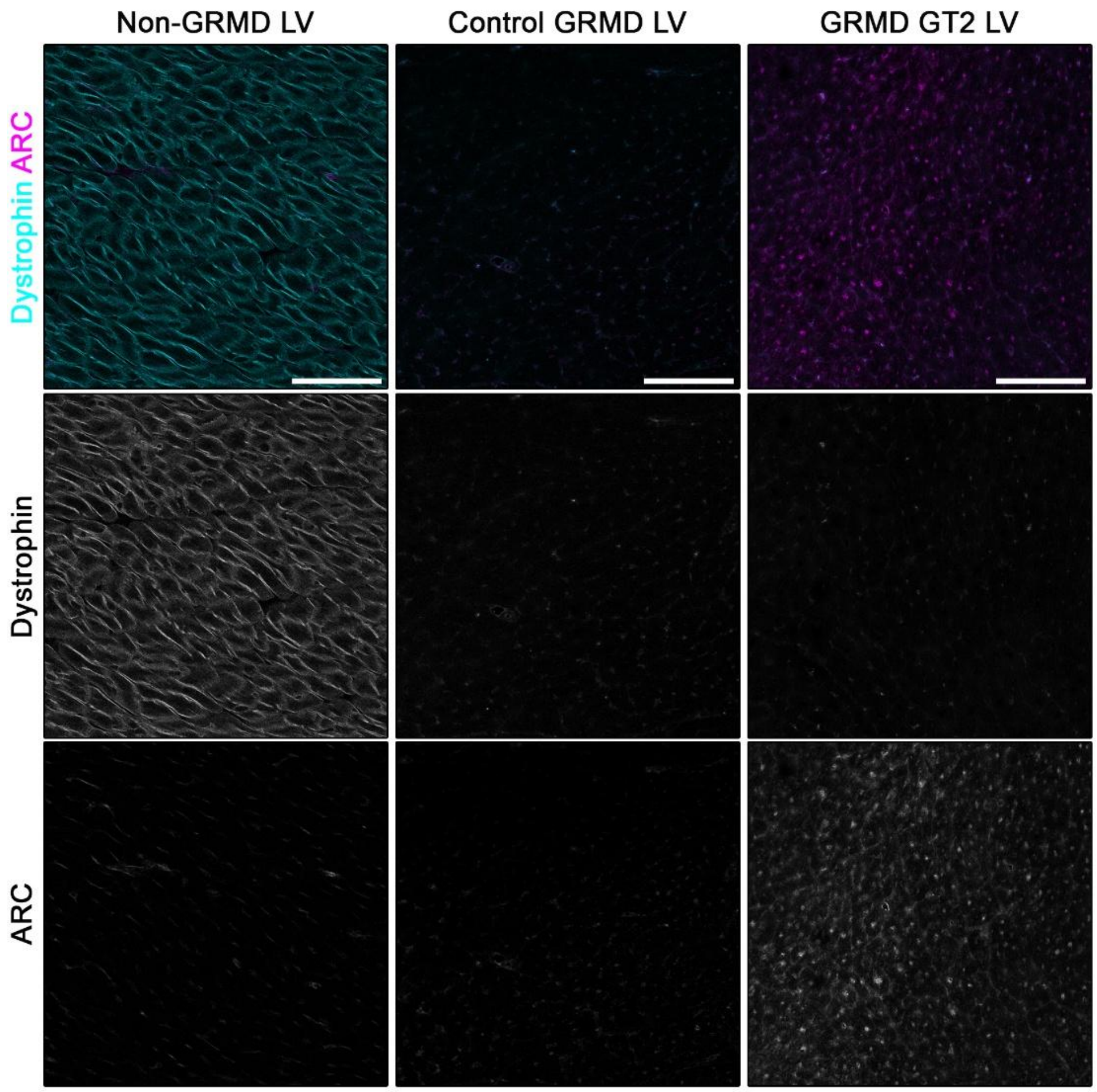

Supplemental Figure 8. Immunofluorescent detection of ARC in treated GRMD hearts. Left ventricle (LV) sections from non-GRMD, control GRMD, and S100A1-ARC treated GRMD (GT2) hearts were stained for immunofluorescent detection of dystrophin and ARC. Scale bars represent 100 µm.

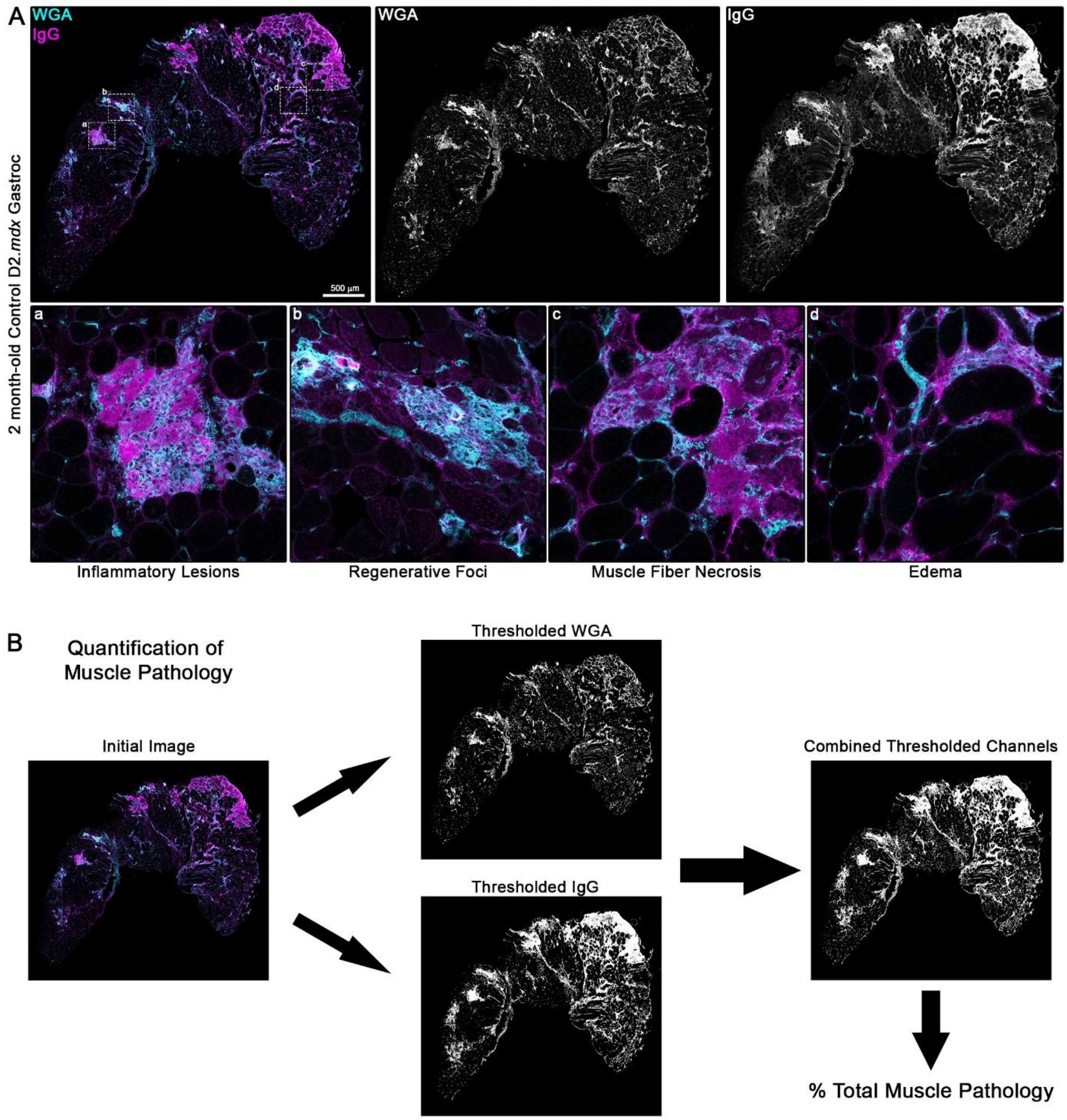

Supplemental Figure 9. Quantification of muscle pathology in D2.*mdx* skeletal muscle. (**A**) Representative wheat germ agglutinin (WGA) and endogenous mouse IgG fluorescent staining in 2 month-old control D2.*mdx* gastrocnemius reveals different features of skeletal muscle pathology, including inflammatory lesions, regenerative foci, myofiber necrosis, and edema (Scale bar = 500 µm). (**B**) Confocal microscopy images of entire muscle sections were analyzed for estimated total muscle pathology by thresholding WGA and IgG channels individually and combining both into a single monochromatic image to quantify total muscle pathology as a percentage of the total muscle cross-sectional area.
