# Supplementary material for: Combination S100A1 and ARC gene therapy as a treatment for Duchenne muscular dystrophy cardiomyopathy
Source: JCI Insight. 2026 Jun 9;11(15):e204852. doi: 10.1172/jci.insight.204852 (PMC13464013; doi:10.1172/jci.insight.204852)

Full unedited blot (FUB) for Supplemental Figure 4

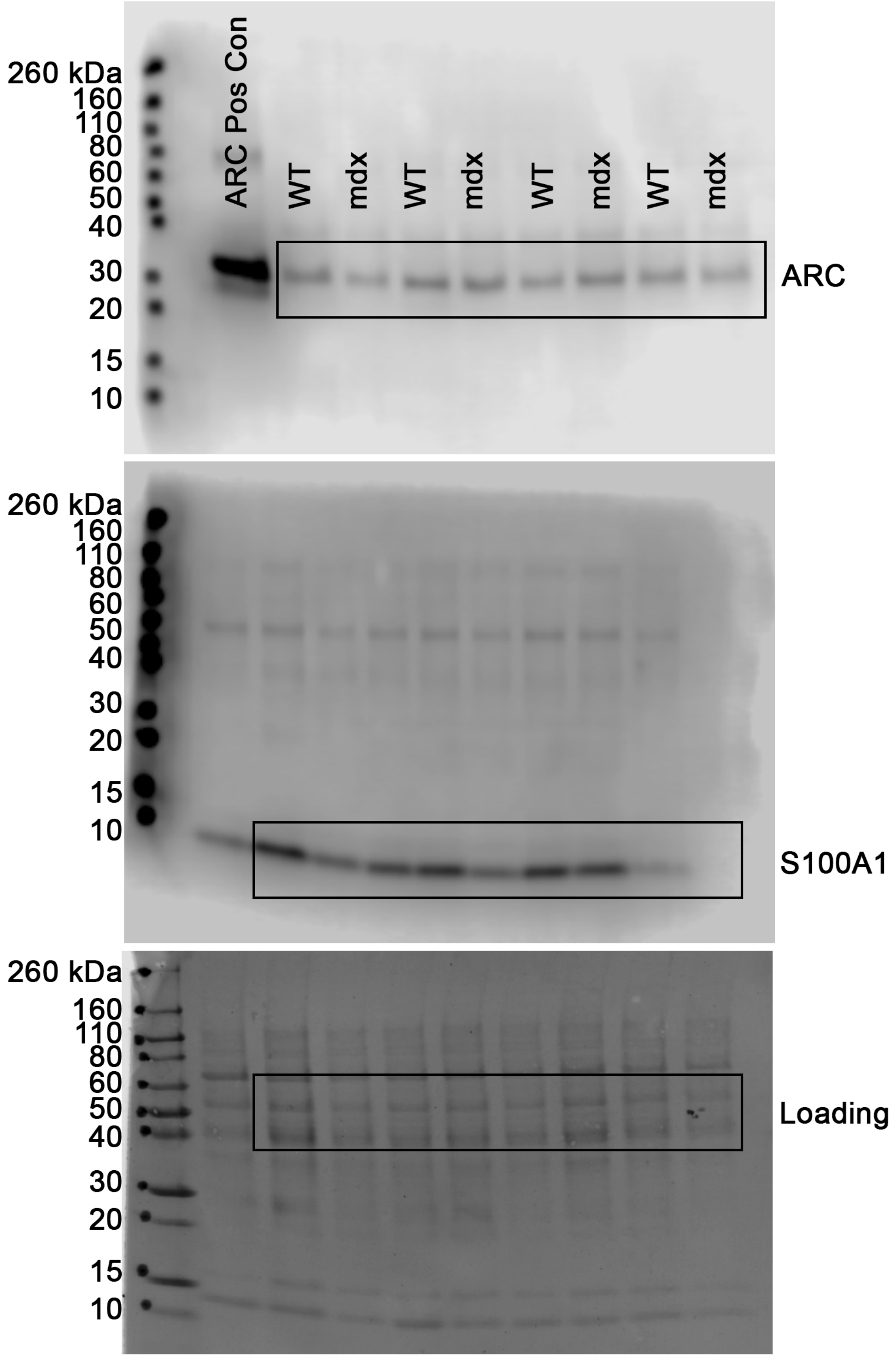

FUB for Figure 4B

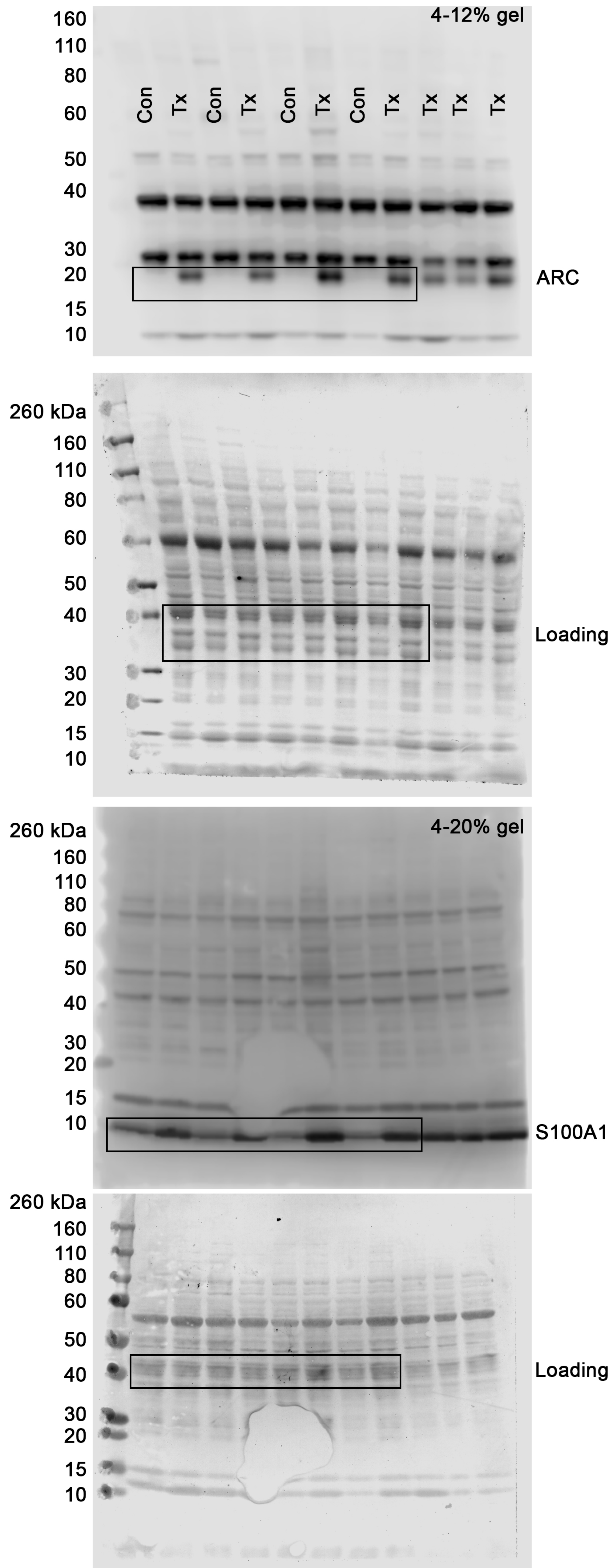

FUB for Supplemental Figure 6E

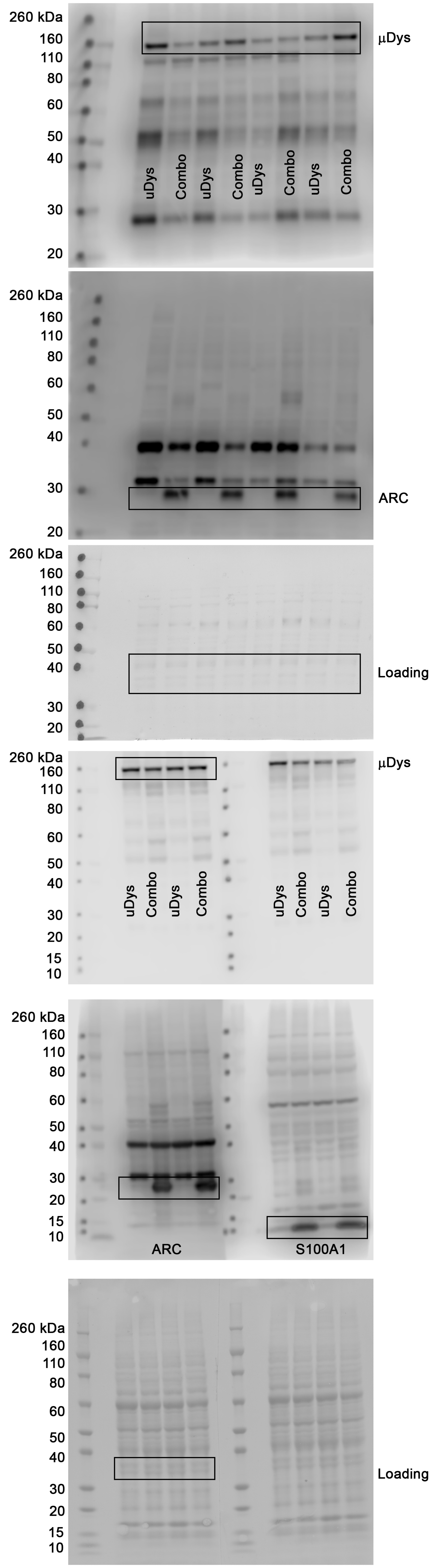

FUB for Supplemental Figure 7B

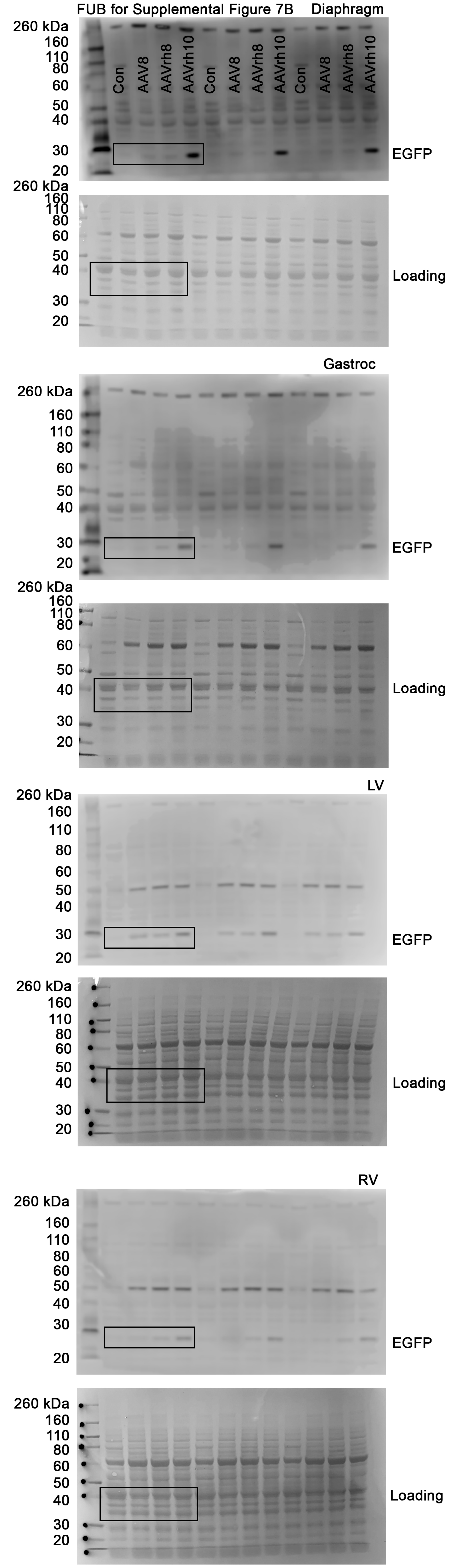

Supplement: Supplementary file 2 [file jciinsight-11-204852-s250.pdf]
